# Two *Nucleoporin98* homologous genes jointly participate in the regulation of starch degradation to repress senescence in *Arabidopsis*

**DOI:** 10.1186/s12870-020-02494-1

**Published:** 2020-06-26

**Authors:** Long Xiao, Shanshan Jiang, Penghui Huang, Fulu Chen, Xu Wang, Zhiyuan Cheng, Yuchen Miao, Liangyu Liu, Iain Searle, Chunyan Liu, Xiao-Xia Wu, Yong-Fu Fu, Qingshan Chen, Xiao-Mei Zhang

**Affiliations:** 1 Key Laboratory of Soybean Biology, Ministry of Education/College of Agriculture, Northeast Agricultural University, Harbin, 150030 China; 2 MOA Key Lab of Soybean Biology (Beijing), National Key Facility of Crop Gene Resource and Genetic Improvement, Institute of Crop Sciences, Chinese Academy of Agricultural Sciences, Nandajie 12, Zhongguancun, Haidian District, Beijing, 100081 China; 3 Key Laboratory of Plant Stress Biology, State Key Laboratory of Cotton Biology, School of Life Sciences, Henan University, Kaifeng, 475004 China; 4 College of Life Sciences, Capital Normal University, Beijing, 100048 China; 5 School of Biological Sciences, School of Agriculture, Food and Wine, The University of Adelaide, Adelaide, South Australia 5005 Australia

**Keywords:** Nup98, Starch, Sugar, Senescence, Nuclear pore complex, Nucleoporin

## Abstract

**Background:**

Starch is synthesized during daylight for temporary storage in leaves and then degraded during the subsequent night to support plant growth and development. Impairment of starch degradation leads to stunted growth, even senescence and death. The nuclear pore complex is involved in many cellular processes, but its relationship with starch degradation has been unclear until now. We previously identified that two *Nucleoporin98* genes (*Nup98a* and *Nup98b*) redundantly regulate flowering via the *CONSTANS* (*CO*)-independent pathway in *Arabidopsis thaliana*. The double mutant also shows severe senescence phenotypes.

**Results:**

We find that *Nucleoporin 98* participates in the regulation of sugar metabolism in leaves and is also involved in senescence regulation in *Arabidopsis*. We show that *Nup98a* and *Nup98b* function redundantly at different stages of starch degradation. The *nup98a-1 nup98b-1* double mutant accumulates more starch, showing a severe early senescence phenotype compared to wild type plants. The expression of marker genes related to starch degradation is impaired in the *nup98a-1 nup98b-1* double mutant, and marker genes of carbon starvation and senescence express their products earlier and in higher abundance than in wild type plants, suggesting that abnormalities in energy metabolism are the main cause of senescence in the double mutant. Addition of sucrose to the growth medium rescues early senescence phenotypes of the *nup98a-1 nup98b-1* mutant.

**Conclusions:**

Our results provide evidence for a novel role of the nuclear pore complex in energy metabolism related to growth and development, in which *Nup98* functions in starch degradation to control growth regulation in *Arabidopsis*.

## Background

The nuclear pore complex (NPC) is the key bridge for communication of macromolecules between the nucleus and cytoplasm and it regulates gene expression [1]. NPC is built by at least 30 unique nucleoporins (Nups), which are highly conserved in eukaryotic cells [2, 3]. Nup98 is a mobile and peripheral Phe-Gly domain (FG)-containing nucleoporin which spans from the nucleus and cytoplasmic parts of the central channel of the NPC [4, 5]. *Arabidopsis thaliana* Nup98a (also known as DRACULA2, DRA2) is also found in different subcellular locations [6]. Nup98 is involved in the regulation of cargo export and import, gene expression, transcriptional memory, and multiple developmental processes in animals and yeast [4–9]. In *Arabidopsis*, *DRA2* regulates the shade avoidance syndrome [6]. In rice, the Nup98 homolog APIP12 (AVRPIZ-T INTERACTING PROTEIN12) is involved in basal resistance against the pathogen *Magnaporthe oryzae* and targeted by the *Magnaporthe* effector AvrPiz-t [10]. In a recent report in *Arabidopsis*, the interaction between the proteins Nup98 and Nup88/MOS7 (MODIFIER OF SNC1,7) is required for plant immunity against the necrotrophic fungal pathogen *Botrytis cinerea* and mitogen-activated protein kinase signalling [11]. We found that Nup98 contributes to flowering regulation in *Arabidopsis* [12].

Senescence is an important cellular process associated with various developmental and environmental cues [13]. Before death, plants remobilize resources in senescing organs and translocate them to sink organs to support their growth and development [14–16]. To date, at least 200 genes have been identified that regulate, or participate in, senescence processes in plants. Starch, a main photosynthetic product synthesized in leaves, is degraded at night to support plant development. Impairment of gene function during different steps in starch degradation hinders plant growth to different extents [17, 18]. Sugar directly or indirectly regulates senescence: sugar accumulation not only triggers and accelerates, but may also delay senescence [19–21]. In fact, in response to sugar, senescence process in plants is age- or condition-dependent [20, 22, 23]. Within the sugar pathway, three key genes have been identified: glucose sensor *HEXOKINASE 1* (*HXK*1), energy sensor *PROTEIN KINASE10* (*KIN10*) and *KIN11*, and *TOR* (*THE TARGET OF RAPAMYCIN*) [21, 24–27].

Currently, the mechanism of NPC in regulation of starch degradation and senescence in plants is still unknown. However, in animals there are several studies reporting that NPC controls cell senescence through modifying chromosome structure, DNA repair and replication or cell division [28–32]. Impairment of NPCs results in dysfunction of nucleo-cytoplasm transportation [30]. Both *Nup107* [33] and *Tpr* (*TRANSLOCATED PROMOTER REGION*) [34, 35] have been linked to cancer cell proliferation and cellular senescence in aging cells.

In this study, we focused on two *Nup98* homologous genes, *Nup98a* and *Nup98b,* in *Arabidopsis thaliana*. Single mutants of either of these genes in *Arabidopsis* have no obvious flowering and senescence phenotypes. However, the double mutant, *nup98a-1* and *nup98b-1*, show significant early-senescence phenotypes. Gene expression analysis demonstrates that *Nup98a* and *Nup98b* participate in starch degradation conferring growth regulation. Further analysis suggests that early senescence in the double mutant may result from a defect in the initial steps of starch degradation and result in a dysfunction in energy supply. Interestingly, the early senescence phenotype in this double mutant can be rescued by the addition of sugar to the growth medium. Our data suggest that *Nup98a* and *Nup98b* might function redundantly in regulation of starch degradation and contribute to normal growth and development in *Arabidopsis*.

## Results

### *Nup98* mutation results in early senescence in *Arabidopsis thaliana*

**Fig. 1:**
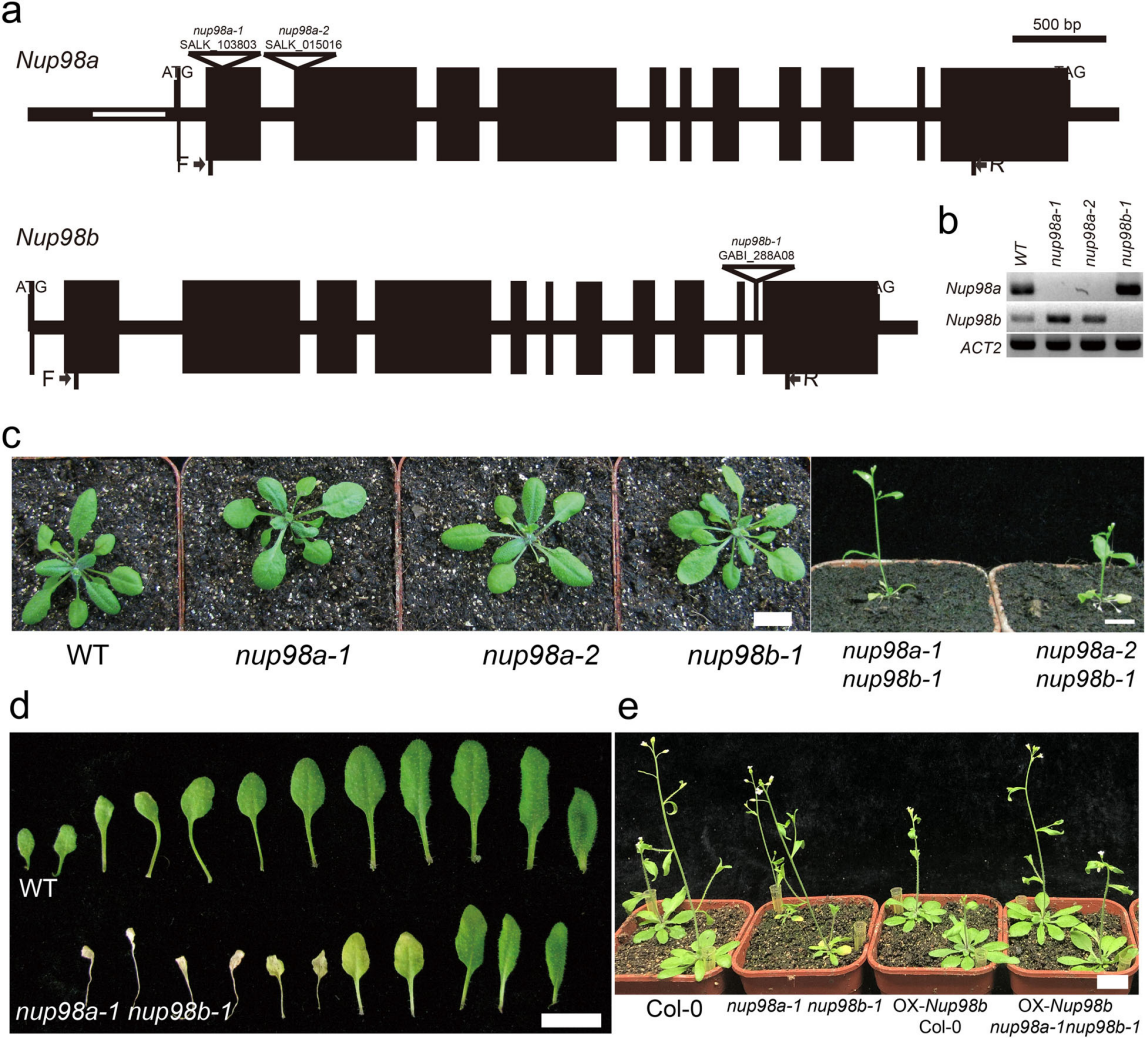
Mutation of *Nup98* leads to an early senescence phenotype. **a**: T-DNA insertion alleles in *Nup98a* and *Nup98b*. Black bars indicate exons, thin black lines indicate introns or UTR, open lines in the 5′-UTR indicates an intron, triangles show T-DNA insertions with the mutant name and T-DNA identifier above the symbol. **b**: RT-PCR confirmed the single mutants of *nup98a-1*, *nup98a-2*, and *nup98b-1*. The position of forward (F) and reverse (R) primers are indicated in **a** (black arrows). *ACT2* was used as a control. **b** refers to the full-length image being available in Figure SI. **c**: Single mutants of *nup98a-1*, *nup98a-2*, and *nup98b-1* displayed similar phenotypes to wild type plants. However the double mutants of *nup98a-1 nup98b-1* and *nup98a-2*, *nup98b-1* showed severe early senescence. The photographs were taken on day 20 after germination. **d**: The leaf senescence phenotype of the *nup98a-1 nup98b-1* double mutant rosette leaves harvested at inflorescence emergence. **e**: Ectopic expression of *Nup98b* rescued the leaf senescence phenotype of the *nup98a-1 nup98b-1* double mutant. All plants were grown in soil from germination. The photographs were taken on day 30 after germination. An * indicates measurements that were significantly (*P < 0.05 or **P < 0.01) different from the control. Error bars indicate ± SD of the mean. All the images are our own data

Nup98 is a highly conserved nuclear pore protein in eukaryotes. In *Arabidopsis thaliana*, there are two homologs of mammalian *Nucleoporin98* - *Nup98a* (At1g10390) and *Nup98b* (At1g59660), which share highly conserved amino acid sequences in the Phe-Gly (FG)-repeats and autoproteolytic domain (APD, Supplementary Figure 1) [3, 6]. *Nup98a* was previously reported as *DRACULA2* (*DRA2*), a regulator of the shade avoidance syndrome (SAS) in *Arabidopsis* [6] and immune responses to a rice fungal pathogen [11]. To investigate *Nup98* functions in plant development, we screened mutants of *nup98a* (SALK_080083, SALK_090744, SALK_023493, SALK_103803, SALK_015016 ordered from ABRC) and *nup98b* (CS803848 and GABI_288A08 ordered from GABI T-DNA mutant center). Homozygous lines were isolated for the following insertional mutants of SALK_103803, SALK_015016, GABI_288A08, and among them SALK_103803 and GABI_288A08 were the mutants reported by Parry [9]. The T-DNAs in these homozygous mutants are inserted in protein-encoding regions (Fig. 1a) and RT-PCR demonstrated that these mutants were null alleles (Fig. 1b, Supplementary Figure 2), consistent with Parry’s results [9]. As in previous studies [9], we did not observe any obvious phenotypes in flowering and senescence in either the *nup98a* or *nup98b* single mutants compared to wild type plants under long-day photoperiod conditions (Fig. 1c). As Nup98a and Nup98b share highly conserved amino acid sequences (Supplementary Figure 1), we tested the hypothesis that *Nup98a* and *Nup98b* function redundantly. Strikingly, two double mutants, *nup98a-1 nup98b-1* and *nup98a-2, nup98b-1*, displayed similar early senescence phenotypes when compared with wild type plants (Fig. 1c and d). The senescence phenotype appeared not only in leaves, but also in the whole plant. Also, the double mutant plants had additional phenotypes, including smaller inflorescences, flowers and siliques, short stature, and severe sterility when compared with wild type plants (Supplementary Figures 3 and 4). We recently reported that the *nup98a-1 nup98b-1* double mutants have an early flowering phenotype [12]. Expressing the *Nup98b* gene in the double mutant, rescued the senescence phenotypes (Fig. 1e). These results confirm that *Nup98a* and *Nup98b* act redundantly.

To investigate whether the senescence phenotype is specific to the *nup98a-1 nup98b-1* mutant, we selected another three nucleoporin mutants, *nup96–1*, *nup160–1*, and *nup107–1*, which showed flowering phenotypes in our previous report [36], to analyze the effect of other nuclear pore components on senescence. To our surprise, no early senescence phenotypes were observed in these mutants (Supplementary Figure 5), suggesting that some of nucleoporins may not be involved in the regulation of senescence and that *Nup98* may have specific functions in senescence regulation.

### *The Nup98* gene is involved in multiple pathways of senescence initiation

**Fig. 2:**
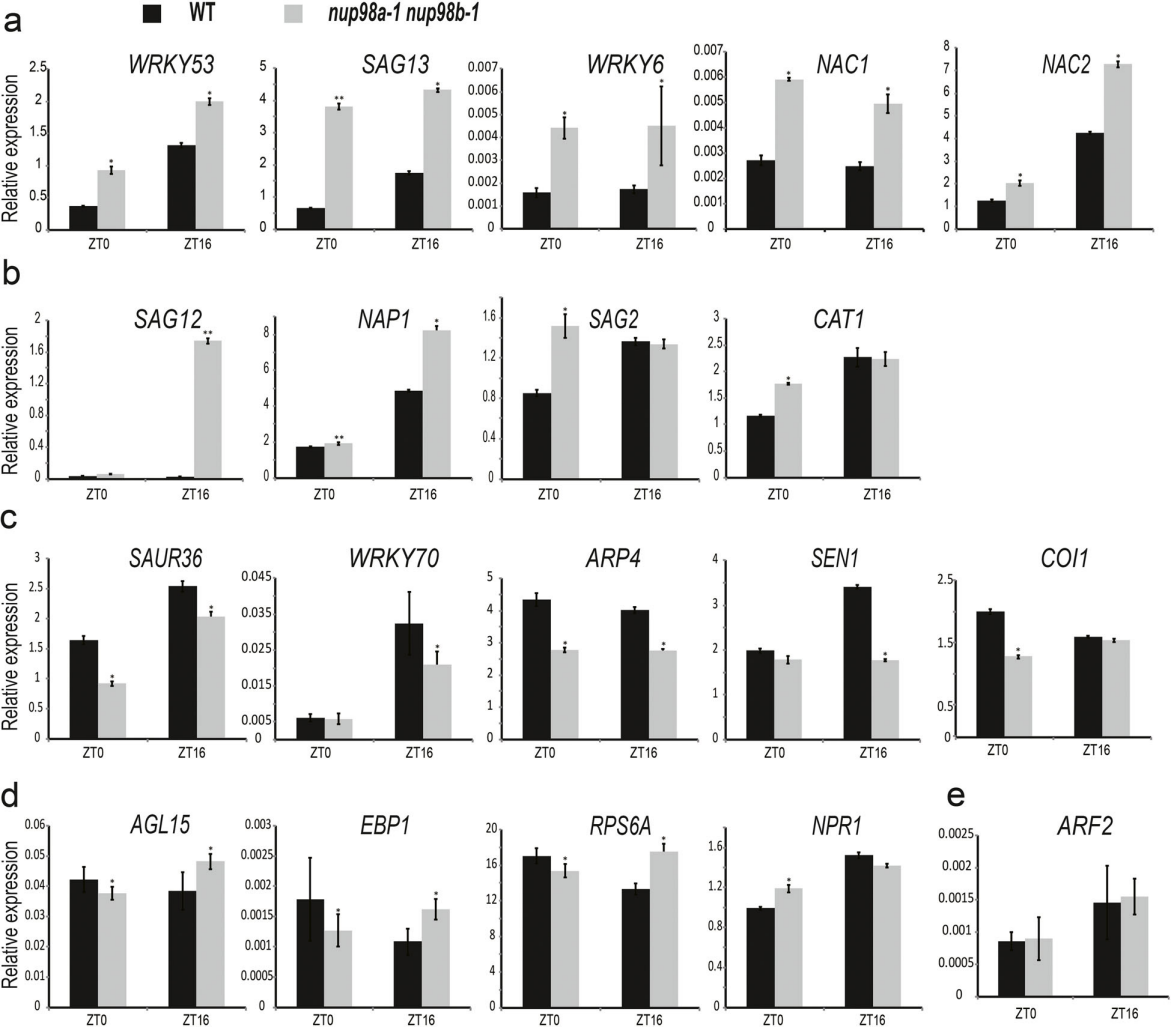
The expression of genes related to senescence are altered in the *nup98a-1 nup98b-1* double mutant. Up-regulated genes both at dawn (ZT0) and dusk (ZT16) (**a**) or at either dawn (ZT0) or dusk (ZT16) (**b**). **c**: Dawn-regulated genes at dawn (ZT0) and/or dusk (ZT16). **d**: Up-regulated or dawn-regulated genes at dawn (ZT0) and dusk (ZT16), respectively. **e**: Constitutively expressed genes. The 14-day-old seedlings of the *nup98a-1 nup98b-1* double mutant and wild type plants in long day conditions were harvested at ZT0 and ZT16. All RT-PCR measurements were repeated at least three times, each time in triplicate. All RT-PCR gene transcript measurements were normalized to the control *TIP41* (At4g34270) and shown as a relative value. Student’s *t* test was employed for statistical analysis. An * indicates measurements that were significantly (*P < 0.05 or **P < 0.01) different from the control. Error bars indicate ± SD of the mean

Because the early senescence observed in the *nup98a-1 nup98b-1* double mutant could be a secondary effect of altered development, we further explored the role of *Nup98a* and *Nup98b* in senescence regulation. To date, at least 200 genes have been identified to participate in senescence regulation in plants [37]. We summarized main literatures as shown in Supplementary Figure 6, which shows that various endogenous and environmental cues, such as hormones, sugar, light and photoperiod, and stresses, trigger plant senescence in multiple cross-talking patterns. To identify the potential link to early senescence, we measured mRNA abundance of important senescence-associated genes in the *nup98a-1 nup98b-1* double mutant plants (Supplementary Figure 6) at ZT0 (Zeitgeber 0, the time when light turned on) and ZT16 (the time when light turned off) in plants grown under long day conditions by real time quantitative PCR (RT-qPCR). As shown in Fig. 2, gene expressions in the *nup98a-1 nup98b-1* double mutant were significantly different from that in wild type plants. In the first category, *WRKY53* (encoding WRKY DNA-BINDING PROTEIN53), *SAG13* (encoding SENESCENCE-ASSOCIATED GENE13), *WRKY6*, *NAC1* (encoding NAM, ATAF, and CUC) and *NAC2* displayed higher transcript abundances at both ZT0 and ZT16 (Fig. 2a), suggesting that genes in the stress pathway (*WRKY53*, *WRKY6*, and *NAC1*) and SA pathway (*WRKY53*, *WRKY6*, and *NAC2*) were related to the senescence phenotypes of the *nup98a-1 nup98b-1* double mutant. In the second category, *SAG12*, *NAP* (encoding *NAC DOMAIN CONTAINING PROTEIN*), *SAG2* and *CAT1* (encoding *CATALASE1*), also genes in stress and SA pathways, were only increased in abundance at either ZT0 or ZT16 (Fig. 2b). In contrast, in the third category, *SAUR36* (encoding *SMALL AUXIN UPREGULATED36*), *WRKY70*, *ARP4* (encoding *ACTIN-RELATED PROTEIN4*), *SEN1* (encoding *SPLICING ENDONUCLEASE1*), and *COI1* (encoding *CORONATINE INSENSITIVE1*) were decreased in abundance at either ZT0 and/or ZT16 (Fig. 2c), indicating that auxin (*SAUR36*) and jasmonate (*COI1*) signalling may be negatively related to the *nup98* senescence phenotypes. The abundance of *AGL15* (encoding *AGAMOUS-LIKE15*), *EBP1* (encoding *ERBB-3 BINDING PROTEIN1*), *RPS6a* (encoding *RIBOSOMAL PROTEIN6a*), and *NPR1* (encoding *NONEXPRESSOR OF PR-GENES1*) had the opposite changes at ZT0 and ZT16 (Fig. 2d), suggesting that the function of these genes on *Nup98* senescence regulation may be dependent on the circadian clock.

This expression profile suggests that there are at least three senescence processes affected in the *nup98* double mutant. Firstly, several pathways, mainly the stress and SA pathways, were involved in regulation of senescence in the *nup98a-1 nup98b-1* double mutant. Secondly, different genes function in their own special modes by being positively or negatively regulated at different phases (morning or afternoon phases). Thirdly, some genes, such as *WRKY53*, *WRKY6*, *NAP1*, and *SAG2*, may play roles in multiple pathways. The results indicate that the senescence phenotypes of the *nup98* double mutant involve multiple pathways. Many genes showed circadian expression patterns, consistent with our previous report that *Nup98* genes are regulated by processes involving the circadian clock [12].

### Starch metabolism is impaired in the *nup98a-1 nup98b-1* double mutant

**Fig. 3:**
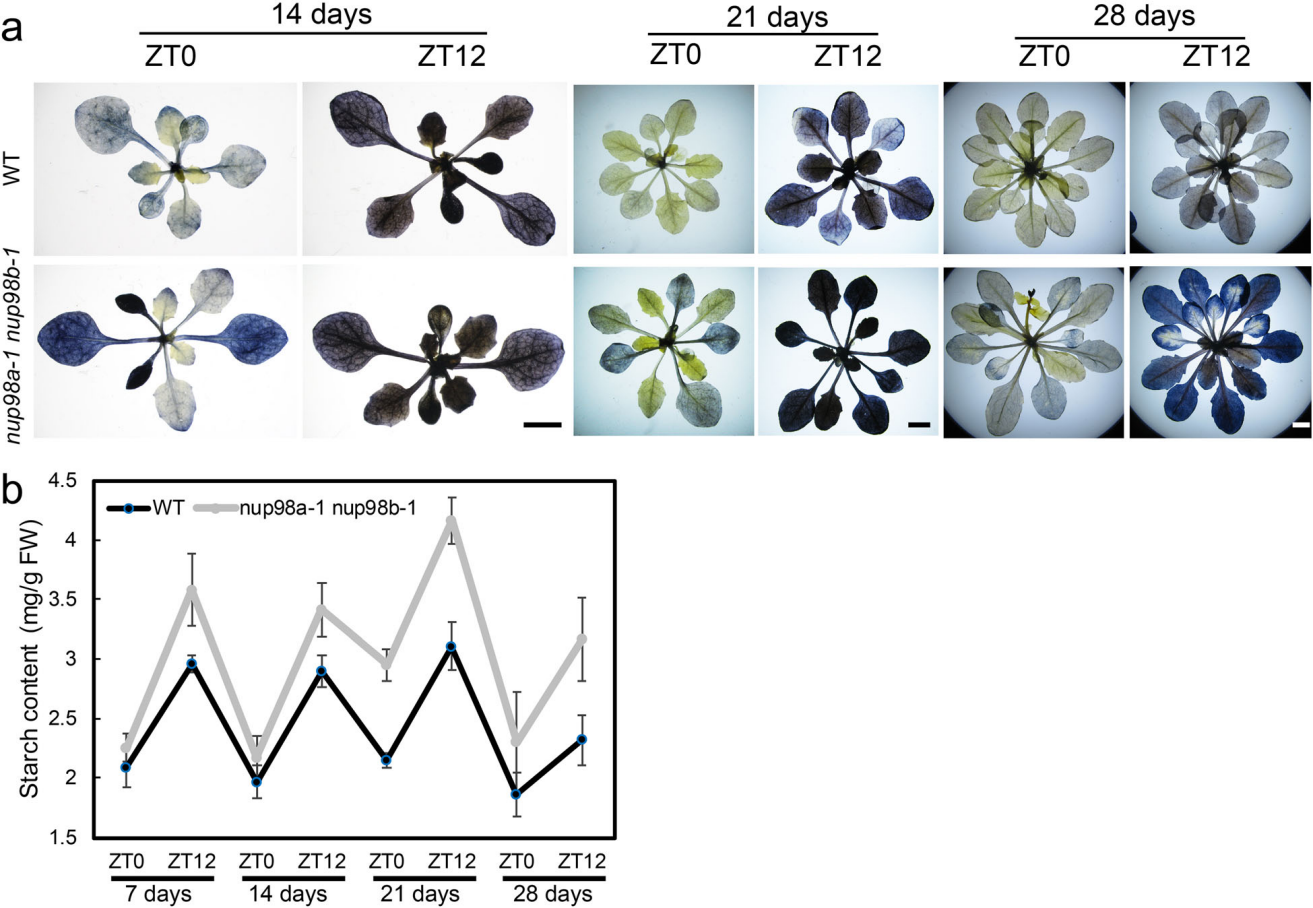
The *nup98a-1 nup98b-1* double mutant has abnormal starch degradation. *Arabidopsis* seedlings were grown on 1/2 MS medium in 2 h light/12 h dark conditions after treatment at 4 °C for 3 days. Plants were grown for an additional 7, 14, 21, or 28 days and were harvested at ZT0 and ZT12 for starch staining (**a**) and starch quantitative assays (**b**) using a spectrophotometric starch determination assay (Solarbio, Beijing, Cat#BC0700). All the images are our own data

During photosynthesis, starch is synthesized and stored in chloroplasts and then degraded at night. A number of genes are involved in starch degradation in plants (Supplementary Figure 7) [17, 18]. Firstly, we checked starch homeostasis in leaves of the *nup98a-1 nup98b-1* double mutant plants grown under 12 h light/12 h dark conditions (at ZT0, dawn, and ZT12, dusk) (Fig. 3). Leaves of all plants accumulated starch at dusk, but double mutant plants had much more starch than wild type plants as determined by both iodine staining (Fig. 3a) and starch quantitative assays (Fig. 3b). The more intense signals in older (21 and 28-day) leaves in the double mutant may be a consequence of accumulating starch over time. Therefore, the double mutant accumulated much more starch, not only at dawn but also at dusk, than wild type plants, and this phenotype was much clearer in 14- and 21-day-olds seedlings (Fig. 3a and b). Together, these results suggest that starch metabolism is impaired in the *nup98a-1 nup98b-1* double mutant.

**Fig. 4:**
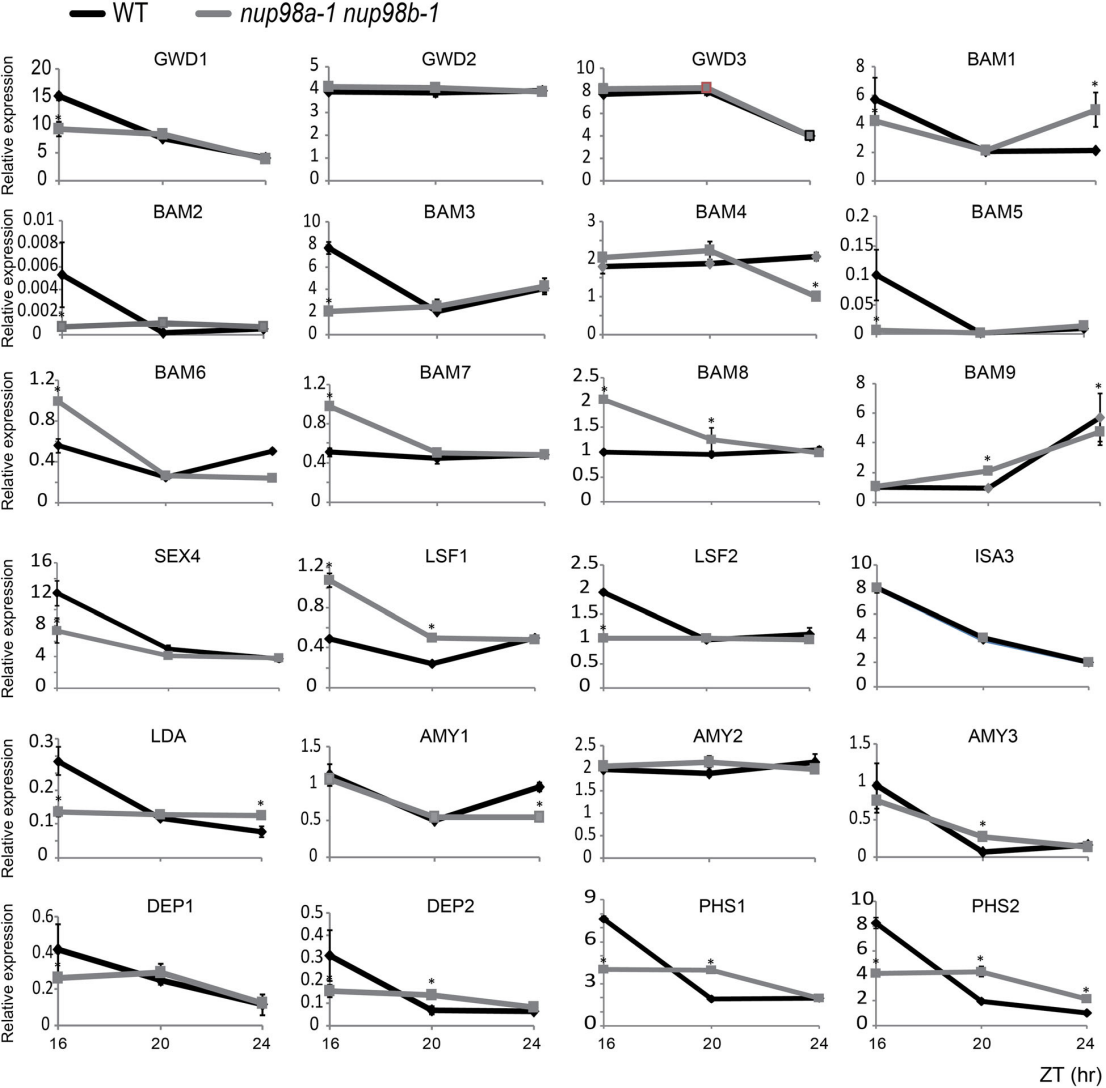
The transcription of genes related to starch metabolism is altered in the *nup98a-1 nup98b-1* double mutant. The growing conditions of seedlings are the same as those in Fig. 3. Samples were harvested at ZT16, 20 and 24 during the dark phase. All RT-PCR measurements were repeated at least three times, each in triplicate. All RT-PCR gene transcription measurements were normalized to the control *TIP41* (At4g34270) and expressed as relative transcription values. Statistical analysis was performed as described in Fig. 2

We measured the transcript abundance of genes involved in starch metabolism [17] by RT-qPCR (Fig. 4) and found that many genes had significantly lower RNA accumulation in the double mutant at least at one time point when compared with wild type plants. These genes encode enzymes for the degrading process of starch, not only in the early steps in chloroplasts [17], such as *GWD1* (encoding α-GLUCAN WATER, DIKINASE1; or STARCH EXCESS1, SEX1), *β*-*BAM1* (encoding β-AMYLASE1), *BAM3*, *BAM5*, *BAM6*, *BAM7*, *BAM8*, *SEX4*, and *LSF1* (encoding LIKE SEX FOUR1), but also in the later steps, such as *LDA* (encoding LIMIT-DEXTRINASE*)*, *AMY1* (encoding α-AMYLASE) and *AMY2*, *DPE1* (encoding DISPROPORTIONING ENZYME) and *DPE2*, *PHS1* (encoding α-GLUCAN PHOSPHORYLASE) and *PHS2*. Time-dependent low-expression of these genes suggests that they are under circadian control and *Nup98* itself is involved in circadian regulation in *Arabidopsis* [12]. These genes function at different steps of starch degradation [17]. GWD1 is α-glucan water dikinase, which phosphorylates glucosyl residues of amylopectin at the C-6 position. BAMs are a family of *β*-amylases breaking down the *α*-1,4-linked glucose chains. LSF1 and SEX4 release the phosphate bound at C-6 and C-3 of glucosyl residues. Both ISA (ISOAMYLASE 3) and LDA (LIMIT-DEXTRINASE) hydrolyze *α*-1,6 branch points but have different substrates. AMYs act on *α*-1,4-linkages releasing linear *α*-1,4-linked oligosaccharides and branching *α*-1,4- and *α*-1,6-linked oligosaccharides. DPEs (DISPROPORTIONATING ENZYMEs) transfer glucose/α-1,4-linked glucan moiety from a donor glucan to an acceptor, releasing the non-reducing end of glucose/glucan moiety. PHSs (α--GLUCAN PHOSPHORYLASEs) act on the non-reducing end of *α*-1,4-linked glucose. Lower abundance of these genes resulted in a starch-excess phenotype in the *nup98a-1 nup98b-1* double mutant (Fig. 3) as *sex4* mutants [17]. The mRNA abundance of some other genes involved in starch metabolism, including *GWD2*, *GWD3*, *ISA3* (*ISOAMYLASE3*) and *AMY3*, was not significantly affected, suggesting the effect of *Nup98a/b* on starch metabolism is limited to some specific enzymes.

We also measured the mRNA abundance of genes related to photosynthesis and sugar metabolism by RT-qPCR in the *nup98a-1 nup98b-1* double mutant and wild type plants (Supplementary Figure 8). In terms of photosynthesis-related genes, the decrease of mRNA abundance of *LHCA* (*PHOTOSYSTEM I LIGHT HARVESTING COMPLEX GENE A*) and *LHCB* (*PHOTOSYSTEM I LIGHT HARVESTING COMPLEX GENE B*) was observed in the *nup98a-1 nup98b-1* double mutant at different time points, e.g., *LHCA1/2* and *LHCB1.1* at ZT0 and *LHCA1* and *LHCB1.4* genes at ZT16. We also observed a decrease in mRNA abundances of *KIN10* and *KIN11* in the double mutant when compared to wild type plants, which is expected since both of genes are sugar signaling genes. Both these genes are involved in delaying plant senescence [21, 26, 27], such that their reduced transcript abundance may be associated with earlier senescence (Supplementary Figure 6). We also observed slightly increased mRNA abundance of *HXK1* at dusk (ZT16) that may contribute to earlier senescence via the cytokinin signaling pathway [24]. Unexpectedly, mRNA abundance of *TPS1,* a senescence activator [20], was reduced in the *nup98a-1 nup98b-1* double mutant compared with wild type plants, suggesting that T6P (trehalose-6-phosphate) was not related to the observed senescence. The results indicate that starch synthesis and sugar signalling are impaired in the double mutant.

### Exogenous sugar rescues the early senescence phenotype in the *nup98a-1 nup98b-1* double mutant

**Fig. 5:**
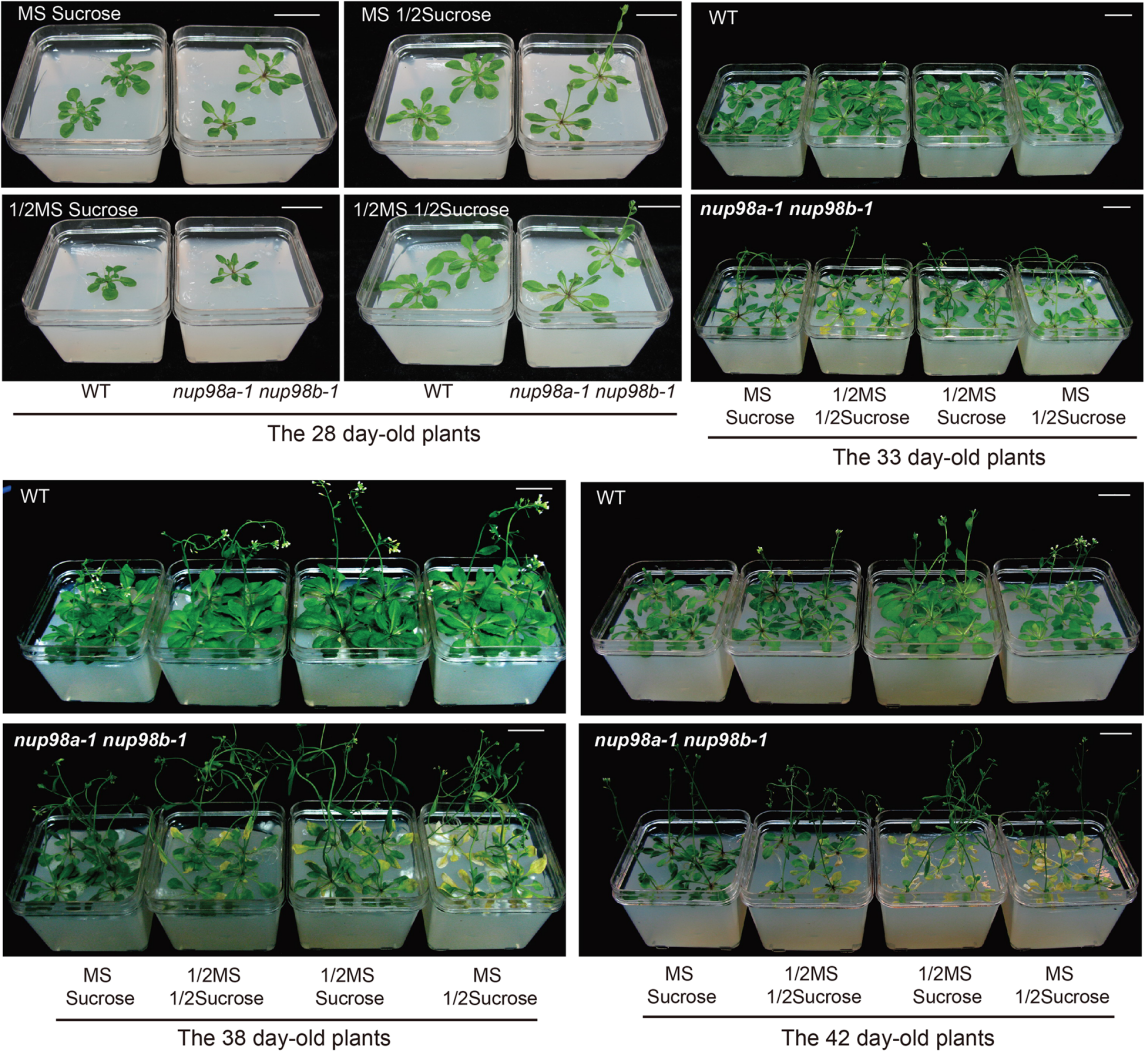
Increased nutrients and sucrose delays senescence in *nup98a-1 nup98b-1* double mutants. The growing conditions of seedlings are same as those in Fig. 3. 1/2MS indicates 1/2 strength of MS. 1/2 sucrose indicates 1.5% sucrose. After stratification, plants were grown under long day conditions. Increases in basal nutrients and sucrose both delayed senescence in the *nup98a-1 nup98b-1* double mutant plants. Bars = 5 cm. All the images are our own data

Our results suggested that the carbon or energy supply was impaired in *nup98a-1 nup98b-1* double mutant plants. We tested the idea by supplying exogenous carbon in the form of sucrose in the growing medium to see if the early senescence phenotype could be rescued. Our results showed that sucrose and MS basal nutrients supported good plant growth even though it was weaker than wild type plants (Fig. 5). Both plants completed their life cycles on medium supplemented with sucrose and basal nutrients. Next, we allowed double mutant and control plants to grow in MS medium until inflorescence emergence and then transferred them to soil. As expected, the double mutant grew as well as wild type plants on MS medium before transplanting (Supplementary Figure 9). However, after transferring to soil, senescence symptoms quickly appeared at day 6 on mutant plant leaves, and the mutant plants wilted at day 30 (Supplementary Figure 9). Wild type plants and the *nup98* double mutant would not survive as plants grown on agarose medium without any supplements (Supplementary Figure 10).

To rule out the possible effect of soil on the senescence phenotypes observed above, we tested whether exogenous macro and micronutrients would complement the phenotypes observed in the double mutant by continuously growing plants on medium at different strengths of sucrose and macro- and micro-nutrients (Supplementary Figure 10). To our surprise, not only did sucrose suppress the early senescence phenotype in the double mutant but also macro- and micro-nutrients in the presence of sucrose. The lower strength basal nutrients (½ MS) enhanced the lower sucrose (1.5%) effect on suppressing senescence, suggesting that both energy supply and basal nutrient metabolism are impaired in the *nup98a-1 nup98b-1* double mutant.

### Starvation and senescence marker genes express highly in the *nup98a-1 nup98b-1* double mutant

**Fig. 6:**
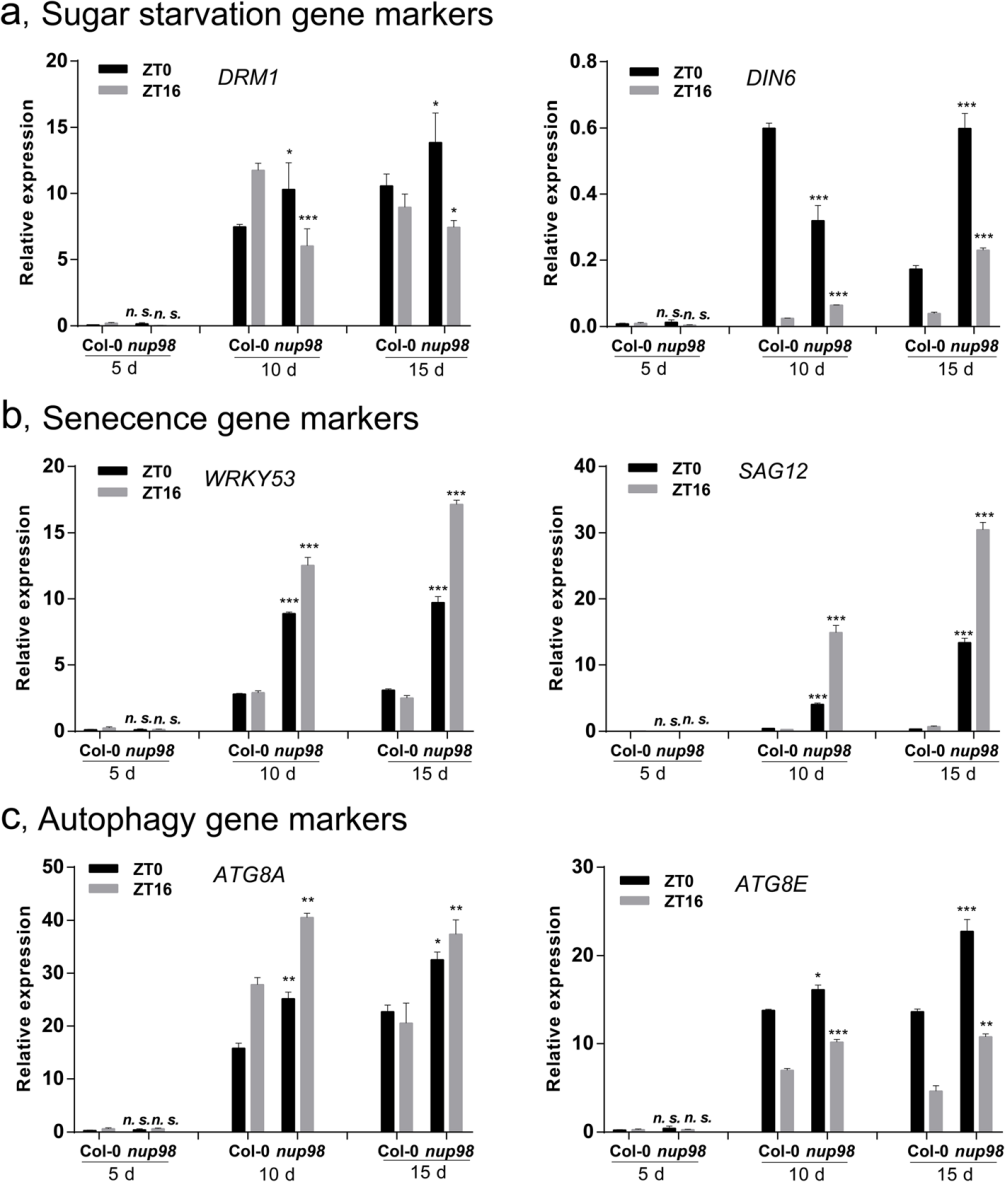
Marker genes of starvation and senescence show altered transcript accumulation in the *nup98a-1*, *nup98b-1* double mutant. **a**: Sugar starvation genes. **b**: Senescence genes. **c**: Autophagy genes. The growth conditions of seedlings are the same as those in Fig. 3. After low temperature treatment for 3 days, the seedlings were grown for 7 days under long day conditions, then transplanted into soil. Samples were harvested on day 5, 10, and 15 at both ZT0 and ZT16. Triplicate qPCR measurements were repeated at least three times, normalized to the reference gene *TIP41* (At4g34270) and expressed as relative transcript accumulation values. Statistical analysis as described in Fig. 2

Results above imply that the *nup98a-1 nup98b-1* double mutant may suffer from sugar starvation and senescence. Therefore, we asked if markers genes of starvation or senescence expressed highly in the double mutant. *DORMANCY-ASSOCIATED PROTEIN-LIKE 1* (*DRM1/DYL1*, At1g28330) and *DARK INDUCIBLE 6* (*DIN6*, At3g47340) [38, 39] are two well-studied sugar starvation marker genes, whereas *SAG12* (At5g45890) and *WRKY53* (At4g23810) are well-characterized senescence markers [40–42]. Autophagy is an important event occurring during sugar starvation and senescence [38, 43, 44], and *AUTOPHAGY8a* (*ATG8a*, At4g21980) and *ATG8e* (At2g45170) are two typical molecular indicators for autophagy in plants [45]. Therefore, we investigated expression changes of these genes in the *nup98a-1 nup98b-1* double mutant compared with those in wild type plants, and the results showed that they had different changes in a time- and developmental-dependent mode (Fig. 6). In the double mutant, *DRM1* began significantly higher expression at ZT0, but lower at ZT12 at a very early stage (day 5 after germination) (Fig. 6a). *DIN* is a light-repressed and dark-induced gene [46], and its high level of expression at ZT0 in the double mutant became obvious at day 15, but at ZT12 higher abundancy appeared earlier from day 10 (Fig. 6a). Compared with wild type plants, the senescence marker *WARKY53* in the double mutant expressed higher at the early stage when *DRM1* expression was in disorder (day 5) (Fig. 6b). *SAG12* is a developmentally-controlled indicator for the later stage of senescence [47, 48]. We found that there was not much difference in *SAG12* expression at the early stage (day 5) between the double mutant and wild type plants. However, *SAG12* had a higher transcript level in the double mutant at both ZT0 and ZT12 from day 10 (Fig. 6b). The two markers of autophagy, *ATG8a* and *ATG8e*, also had higher abundancy of mRNA in most samples of the double mutant from day 10. Taken together, our results showed that the *nup98a-1 nup98b-1* double mutant presented signs of energy starvation, at least at the molecular level, during early developmental stages when plants did not display visible senescence phenotypes. These expression changes had circadian and developmental characters. A previous report shows that different sugars (such as sucrose, glucose, and fructose) have different effects on the regulation of senescence [39]. Therefore, the observance of early and high expression of the marker genes was in accord with senescence characteristics of the *nup98a-1 nup98b-1* double mutant.

### Nup98 proteins mainly localize to both the nuclear membrane and nucleoplasm

**Fig. 7:**
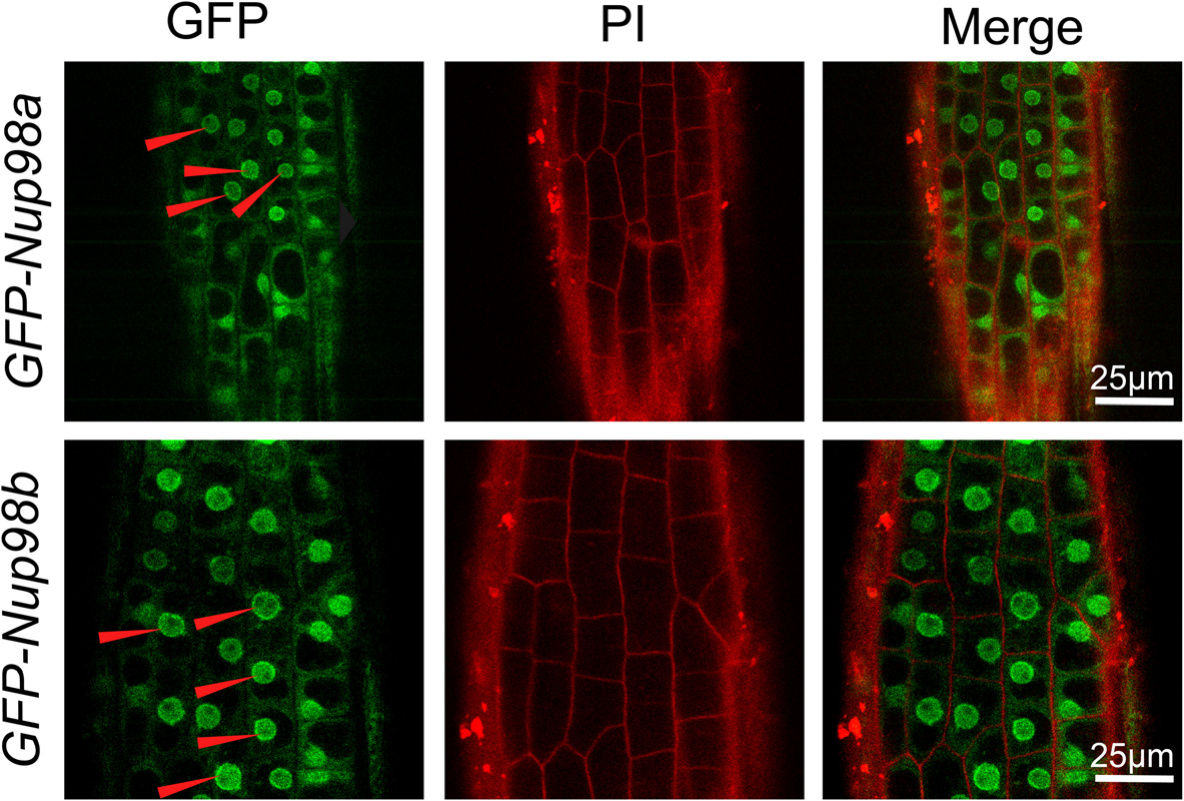
Nup98a and Nup98b proteins are localized to the nuclear membrane and in the nucleoplasm. The green fluorescent protein (GFP) was fused to either Nup98a or Nup98b to generate N-terminal translational fusional proteins, GFP-Nup98a and GFP-Nup98b. Integration of gene constructs driven by the CaMV *35S* promoter gave rise to stably-transformed *Arabidopsis thaliana* plants. Arrow heads indicate that both GFP-Nup98a and GFP-Nup98b were enriched near the nuclear periphery when compared to the cytoplasm. PI (propidium iodide) was used for cell wall staining. All the images are our own data

Nup98 is one of the mobile and peripheral FG (Phe-Gly domain) nucleoporins which is located at both the nuclear and cytoplasmic sides of the NPC central channel [4, 5]. *Arabidopsis* Nup98a (also known as DRA2) is also found in different subcellular compartments [6]. We constructed transgenic *Arabidopsis* plants expressing *35S::GFP:Nup98a* and *35S::GFP:Nup98b* and analyzed the subcellular localization of both fusion proteins. Not surprisingly, both proteins were distributed in the cytoplasm, in the nucleoplasm and at the nuclear periphery (Fig. 7). We observed no significant difference in the subcellular distribution of the two proteins and this is consistent with our observations of their genetic redundancy. In conclusion, our combined results demonstrate that Nup98b proteins localize in both the nucleus and cytoplasm as do Nup98a proteins [6] and their homologs in other organisms [4, 5].

## Discussion

Senescence is a process that is integral to growth and development during the plant life cycle, eventually leading to cell and tissue disintegration and death. In such a physiological process, various basal nutrients are redistributed from senescing organs, such as leaves, to reproductive organs and seeds [13]. However, premature senescence is likely to lead to organ failure or even whole plant death [49]. Fine tuning senescence processes could benefit plants by avoiding the deleterious effect of abiotic stresses and thereby lead to an optimal reproductive outcome. A number of factors, including hormones, developmental age, abiotic stress and light conditions, participate in the regulation of plant senescence [21, 22, 50–59]. While these factors play important and clear roles in plant senescence, the role of sugar is unclear as different research groups have published contradictory results [19–21, 60]. The NPC is an important gatekeeper for both macromolecular transportation between the nucleus and cytoplasm and gene transcription and, therefore, it plays an important role in many different developmental processes in plants [28–32]. Our study provides some novel insights into plant senescence research as we found that the NPC participated in senescence regulation. Our investigation confirmed that *Nup98* genes are involved in starch degradation, conferring senescence initiation in *Arabidopsis*.

*Nup98a* and *Nup98b* function redundantly in senescence regulation in *Arabidopsis*, because the *nup98* single mutants have no obvious senescence phenotypes (Fig. 1), even though a previous report showing longer hypocotyls in the *nup98a* mutant [6]. Similar protein sequences (Figure S1) and subcellular-localization between the Nup98a and Nup98b proteins (Fig. 7) support this hypothesis. Such a senescence phenotype is likely to be specific to mutation of *Nup98* genes, because other nucleoporin mutants detected do not have similar phenotypes (Figure S4). However, it is important to check more mutants of other nucleoporins to elucidate the specificity of *Nup98* functions on senescence regulation. Molecular data support that the *Nup98* genes regulate senescence through multiple pathways, including ethylene, salicylic acid, ABA, cytokinin, and stress, because the marker genes in these pathways, such as *SAG12*, *NAP1*, *WRAY53*, *WRKY6*, *WRKY70*, *NAC1*, *NAC2* and *HXK1*, have significant changes in gene expression (Fig. 2). Additionally, these genes may play a role in a temporal (circadian) manner as their significant expression changes are only at a specific time, at either dawn or dusk (Fig. 2). We recently reported that the expression level of *ELF3* gene and other clock genes significantly reduced in the *nup98a-1 nup98b-1* double mutant compared with that in wild type plants [12]. Therefore, circadian patterns may be involved in the senescence network regulated by the Nup98-encoding genes.

Sugar is confirmed as an important signal in the control of plant growth and development [61]. Many important genes in sugar signaling pathways (*TOR*, *KIN10*, *KIN11*, *TPS1*, *SnRK1*) (Figure S7) and carbon starvation (*DRM1* and *DIN6*) (Fig. 6) were all mis-regulated in *nup98a-1 nup98b-1* double mutant plants. Based on the data presented, sugar availability is likely hindered in the double mutant, suggesting that this might be the main reason for the initiation of senescence (Fig. 5).

In plants, sugar is derived from photosynthesis and stored firstly as starch. Starch accumulated in the chloroplast during the day is broken down into monosaccharides at night [17, 18]. Our evidence showed the *nup98a-1 nup98b-1* double mutant accumulated much higher levels of starch, which was unlikely due to higher photosynthetic efficiency (Figure S7) but impaired starch degradation (Fig.3). This hypothesis was supported by 1) lower transcription levels of genes related to photosynthesis (*LHCA1*, *LHCA2*, *LHCB1.1* and *LHCB1.4*), which would lead to reduced starch synthesis in the double mutant (Figure S7); 2) lower transcript accumulation of starch degradation genes (Fig. 4) controlling many steps in starch degradation (Figure S6) [17] that would lead to starch accumulation in the *nup98a-1*, *nup98b-1* double mutant. Impairment of starch degradation in the double mutant could lead firstly to sugar starvation, subsequently to leaf senescence, and finally to whole plant death.

Beyond this, *Nup98a* and *Nup98b* may also have functions in nutrient metabolism as the concentration of basal nutrients in the growth medium had a significant impact on the growth and senescence in *nup98a-1*, *nup98b-1* double mutants (Fig. 5). In this case, components (sugar or MS) in growth media should be taken into consideration, especially in future senescence studies.

Previous experiments show that the circadian clock regulates starch metabolism in plants [62, 63]. *ELF3* positively regulates starch accumulation, and degradation of starch was significantly slower in *elf3* mutant plants than in the corresponding wild type plants [64]. Therefore, *ELF3* and other clock evening genes, such as *ELF4* and *LUX ARRHYTHMO*, also affect leaf senescence [65, 66]. Many genes studied here showed time-dependent differences in gene expression (Figs. 2, 4 and 6). We previously showed that the expression of many clock genes significantly changed in the *nup98a-1*, *nup98b-1* double mutant [12]. Taken together, clock genes might participate in signalling pathways of *Nup98a/Nup98b*-regulating starch degradation and senescence in *Arabidopsis*.

We recently reported that the *nup98a-1*, *nup98b-1*, *ft-10* triple mutant displays the same late-flowering character as the *ft-10* mutant but it also maintains the early senescence phenotypes of the *nup98a-1*, *nup98b-1* double mutant [12], suggesting that *Nup98a* and *Nup98b* proteins are involved in the regulation of flowering and senescence processes in unrelated or independent pathways.

It is clear that the function of *Nup98* in senescence regulation is indirect. Mutation of *Nup98* genes may lead sequentially to dysfunction of circadian pathways, hinderance of starch degradation, sugar starvation, leaf senescence, and plant death. It would be interesting to determine how *Nup98* controls the function of genes related to starch degradation mediated by circadian clock-related mechanisms.

It is should be pointed that even though *Nup98a* and *Nup98b* function redundantly in the regulation of flowering and senescence, they may play independent and specific functions in other developmental processes, because the *nup98a* single mutant (*dra2*) displays shade avoidance [6].

## Conclusion

NPC has multiple functions in plant development. To the best of our knowledge, our study presents the first report of NPC functions in regulating plant senescence. This result is promising and implies a novel function of NPC in bridging the gap between starch metabolism and senescence control. That is, *Nup98a* and *Nup98b* proteins overlap in their control of starch degradation conferring senescence regulation in *Arabidopsis*.

## Methods

### Plant materials and growth conditions

Seeds of the T-DNA insertion mutants of *nup98a* (SALK_080083, SALK_090744, SALK_023493, SALK_103803, and SALK_015016) and *nup98b* (CS803848 and GABI_288A08) were ordered from ABRC and GABI T-DNA mutant center, respectively. Homozygous screening was according to the protocol provided by SALK (http://signal.salk.edu/). All mutants were identified by Dr. Long Xiao. Except where indicated in the text, *Arabidopsis thaliana* Columbia wild type plants and their derived mutants were grown under long day conditions (16 h/8 h, light/dark) with 100 μmol m^− 2^ s^− 1^ fluorescent lighting. Plants were grown in soil in 10 cm pots or on medium in petri dishes containing different strengths of sucrose and MS (Murashige & Skoog) basal medium (Sigma, #M5524), which contains macronutrients and micronutrients of the original classic formulation (NH_4_NO_3_, 1650.0 mg·L^− 1^; KNO_3_, 1900.0 mg·L^− 1^; CaCl_2_, 332.2 mg·L^− 1^; MgSO_4_·7H_2_O, 180.7 mg·L^− 1^; KH_2_PO_4_, 170.0 mg·L^− 1^; FeSO_4_·7H_2_O, 27.8 mg·L^− 1^; H_3_BO_3_, 6.2 mg·L^− 1^; MnSO_4_·H_2_O, 16.9 mg·L^− 1^; CoCl_2_·6H_2_O, 0.025 mg·L^− 1^; ZnSO_4_·7H_2_O, 8.6 mg·L^− 1^; Na_2_MoO_4_·2H_2_O, 0.25 mg·L^− 1^; KI, 0.83 mg·L^− 1^; CuSO_4_·6H_2_O, 0.025 mg·L^− 1^; Na_2_EDTA·2H_2_O, 37.26 mg·L^− 1^; Glycine (free base), 2 mg·L^− 1^; myo-Inositol, 100 mg·L^− 1^; Nicotinic acid (free acid), 0.5 mg·L^− 1^; Pyridoxine·HCl, 0.5 mg·L^− 1^; Thiamine·HCl, 0.1 mg·L^− 1^) [67].

### Gene and promoter cloning and plasmid construction

Standard GATEWAY (Invitrogen) methods were employed for gene cloning and plasmid construction. Most vectors were developed by our lab [68]. The full-length of *Nup98a* and *Nup98b* open reading frames was PCR-amplified with specific primers (Table S1), and then cloned into Fu30 [68] containing an N-terminal GFP marker. The gene entry vectors (Fu30-*GFP:Nup98a* or Fu30-*GFP:Nup98b*), the *35S* promoter entry vector (Fu76-*35S*) and the binary vector (Fu39–2) [68] were applied to LR reaction (Invitrogen). Both Fu39–2-*35S:GFP:Nup98a* and Fu39–2-*35S:GFP:Nup98b* binary vectors were applied to transform *A. thaliana* using the floral dipping method [69]. Homozygous transgenic lines were used for molecular characterization and phenotype analysis.

### Semi-quantitative PCR, quantitative real time RT-PCR, and subcellular localization

Whole seedlings were harvested at ZT0 (the time point of light on) and ZT16 (the time point of light off) at day 14 after germination. RNA preparation, cDNA synthesis and both quantitative real-time and semi-quantitative RT-PCRs were carried out following Xiao et al. [70], except for the use of *At4g34270* as a reference gene in triplicate [71, 72]. All gene accession numbers and relevant primer sequences are listed in Table S1. GFP fluorescent signals were visualized and captured by confocal microscopy, while propidium iodide (PI) was employed for cell wall staining [68].

### Starch staining and quantification analyses

Starch in leaves of 14–21-, and 28-day-old wild type plants and mutant *Arabidopsis* plants was stained with iodine [73]. Samples were harvested at ZT0 (the time point of light on) and ZT12 (the time point of light off) in a 12 h light/12 h dark cycle. Representative plants were used to build up figures. Starch quantitative analysis was carried out according to the instructions of the Starch Determination Kit (Solarbio, Beijing, Cat#BC0700) on a spectrophotometer (RAYLEIGH, VIS-7220 N; Beijing Beifen-Ruili Analytical Instruments (Group) Co., Ltd) using standard curves which were generated with different concentration of glucose (0.2, 0.1, 0.05, 0.025, 0.0125, 0.00625, 0.003125, 0.00156 mg/mL).

### Statistical analysis

Each experiment had at least three biological replicates and similar results were obtained for each replicate. For figures, one representative plant was selected and photographed. The SPSS software package was used to identify significant difference probability (*P*) levels for all data (*P < 0.05, **P < 0.01, and ***P < 0.001, compared to controls). Error bars represent ± SD of the mean.

## Supplementary information

**Additional file 1: Supplementary Table 1.** Primers used in this study.

**Additional file 2:Figure S1.** Protein domains in Nup98a and Nup98b of *Arabidopsis thaliana*.

**Additional file 3:Figure S2.** The original photograph of the gel in Fig. 1b.

**Additional file 4:Figure S3.** The *nup98a1*, *nup98b1* double mutant showed pleiotropic phenotypes in various organs.

**Additional file 5:Figure S4.** The *nup98a1*, *nup98b1* double mutant showed a senescent and sterile phenotype.

**Additional file 6:Figure S5.** Senescent phenotypes were specific to the *nup98a1*, *nup98b1* double mutant compared with mutants of other nucleoporins.

**Additional file 7:Figure S6.** Molecular network of senescence initiation in plants.

**Additional file 8:Figure S7.** A simplefied pathway of starch degradation in chloroplasts.

**Additional file 9:Figure S8.** Expression analysis of genes related to photosynthesis and sugar metabolism in the *nup98a1*, *nup98b1* double mutant.

**Additional file 10:Figure S9.** Exogenous sucrose reduces early senescence in the *nup98a1*, *nup98b1* double mutant plants.

**Additional file 11:Figure S10.** Phenotype analysis of the *nup98a1*, *nup98b1* double mutant compared with WT grown on different mediums.

## Data Availability

All data generated or analyzed during this study are included in this published article and its supplementary information files.

## Abbreviations

NPC: Nuclear pore complex
Nup: Nucleoporin
CO: CONSTANS
ABA: Abscisic acid
ABI: ABA INSENSITIVE
ACS: Aminocyclopropane-1-carboxylate (ACC) synthase
AGL15: AGAMOUS-LIKE 15
AHK: Arabidopsis histidine kinase
AHP: *Arabidopsis thaliana* histidine phosphotransfer proteins
AMY3: Alpha-amylase-like 3
APD: Autoproteolytic domain
APIP12: AVRPIZ-T INTERACTING PROTEIN12
ARP4: ACTIN-RELATED PROTEIN4
ARF: Auxin response factor
ARR: Arabidopsis response regulator
C2H4: Ethylene
ATG8: Autophagy8
BAM: Beta-amylase
CAT: Catalase
CK: Cytokinin
COI1: Coronatine insensitive1
CWINV: Cell wall invertase
DIN6: Dark inducible6
DRM1/DYL1: Dormancy-associated protein-like1
DPE: Disproportionating enzyme
DRA2: DRACULA2
EBP1: Erbb-3 binding protein
EEL: Enhanced em level
EIN: ETHYLENE INSENSITIVE
ELF: EARLY FLOWERING
ETR: ETHYLENE RESPONSIVE
FG: Phe-Gly domain
G6P: 6-phosphoric acid glucose
GFP: GREEN FLUORESCENT PROTEIN
GLK: GOLDEN 2-LIKE TRANSCRIPTION FACTOR
GWD1: α-glucan water: dikinase
HXK1: Hexokinase1
ISA3: Iso-amylase3
KIN: PROTEIN KINASE
LDA: Limit-dextrinase
LHCA: PHOTOSYSTEM I LIGHT HARVESTING COMPLEX GENE A
LHCB: PHOTOSYSTEM I LIGHT HARVESTING COMPLEX GENE B
LSF1: Like sex four1
N: Nitrogen
NAC: NAM, ATAF, AND CUC
NAP: NAC-LIKE PROTEIN
NPR1: NONEXPRESSER OF PR GENES 1
NYC: NONYELLOW COLORING
MOS7: MODIFIER OF SNC1,7
ORE: ORESARA
PHS: α-glucan phosphorylase
PHYB: PHYTOCHROME B
PIF: PHYTOCHROME INTERACTING FACTOR
PYR/PYL/RCAR: PYRABACTIN RESISTANCE/PYR1-LIKE ORREGULATORY COMPONENT OF ABA RECEPTOR
RPS6A: RIBOSOMAL PROTEIN S6A
RT-qPCR: Real time quantitative PCR
SAG: SENESCENCE-ASSOCIATED GENE
SAS: SHADE AVOIDANCE SYNDROME
SAUR: SMALL AUXIN UPREGULATED
SAUR36: SMALL AUXIN UPREGULATED
SEX4: STARCH EXCESS 4
SGR: STAYGREEN
T6P: Trehalose-6-phosphate
TOR: TARGET OF RAPAMYCIN
Tpr: RANSLOCATED PROMOTER REGION
TPS1: Trehalose-6-phosphate synthase1
WRKY: WRKY DNA-BINDING PROTEIN
ZT: Zeitgeber
