# Supplementary material for: Two Nucleoporin98 homologous genes jointly participate in the regulation of starch degradation to repress senescence in Arabidopsis
Source: BMC Plant Biol. 2020 Jun 26;20:292. doi: 10.1186/s12870-020-02494-1 (PMC7318766; doi:10.1186/s12870-020-02494-1)
Supplement: Supplementary file 1 — Additional file 1: Supplementary Table 1. Primers used in this study. [file 12870_2020_2494_MOESM1_ESM.docx]

**Supplementary Table 1. Primers used in this study.**

| Primer Name | Gene ID | Nucleotide Sequence (5’-3’) | Purpose |
| --- | --- | --- | --- |
| At4g34270-F | At4g34270  Reference gene | gtgaaaactgttggagagaagcaa | qRT-PCR |
| At4g34270-R |  | tcaactggataccctttcgca | qRT-PCR |
| AGL15-F | AT5G13790 | ttccttctaccttcttctc | qRT-PCR |
| AGL15-R |  | agtgacttgtctgctatt | qRT-PCR |
| AMY1-F | At4g25000 | cttggctatgattgatgat | qRT-PCR |
| AMY1-R |  | ttatgcgttacttcttctc | qRT-PCR |
| AMY2-F | At1g76130 | ctcggttggagaatgttg | qRT-PCR |
| AMY2-R |  | ctgattatacgctgtctatgg | qRT-PCR |
| AMY3-F | At1g69830 | tgagatattatgccaaggt | qRT-PCR |
| AMY3-R |  | gaacagtgaatccaagtg | qRT-PCR |
| ARF2-F | At5g62000 | gatgatgataaggttgac | qRT-PCR |
| ARF2-R |  | aatctgtgtaagtaggtt | qRT-PCR |
| ARP4-F | [AT1G18450](http://www.arabidopsis.org/servlets/TairObject?id=29135&type=gene) | gtctgagtatgaggaaca | qRT-PCR |
| ARP4-R |  | ctaataatcttcaaggaacaac | qRT-PCR |
| BAM1-F | At3g23920 | accattgaagaacagagaa | qRT-PCR |
| BAM1-R |  | tcatcacaaacacaggaa | qRT-PCR |
| BAM2-F | At4g00490 | agattcaggttgttatgt | qRT-PCR |
| BAM2-R |  | aagtagatgtcaggattg | qRT-PCR |
| BAM3-F | At4g17090 | ggaatagcgagtatggaa | qRT-PCR |
| BAM3-R |  | ctgaagataggagttggt | qRT-PCR |
| BAM4-F | At5g55700 | tgatgatgccgattgatac | qRT-PCR |
| BAM4-R |  | gctaacttgagtgccttaa | qRT-PCR |
| BAM5-F | At4g15210 | aagataccgacaatacag | qRT-PCR |
| BAM5-R |  | gcaacttctataccttct | qRT-PCR |
| BAM6-F | At2g32290 | gtcatcacgaacgataacg | qRT-PCR |
| BAM6-R |  | accatcactccatctacttg | qRT-PCR |
| BAM7-F | At2g45880 | tggatgtgaaggaagaag | qRT-PCR |
| BAM7-R |  | actcgtcattactactactg | qRT-PCR |
| BAM8-F | At5g45300 | cagagatggttgtatgagat | qRT-PCR |
| BAM8-R |  | aggagaaggttgacgata | qRT-PCR |
| BAM9-F | At5g18670 | cttccgttagacacagtt | qRT-PCR |
| BAM9-R |  | ctcaataccttctacaccaa | qRT-PCR |
| CAT1 | At1g20630 | tattcttcgtccgtgatg | qRT-PCR |
| CAT1 |  | cagtatcctccagttctc | qRT-PCR |
| COI1-F | AT2G39940 | atggcggtgtatgtctcag | qRT-PCR |
| COI1-R |  | gcggaagtcacagaggtt | qRT-PCR |
| DPE1-F | At5g64860 | gcaaggagttgatataatgg | qRT-PCR |
| DPE1-R |  | cgctaacaagaagaggaa | qRT-PCR |
| DPE2-F | At2g40840 | ggacttagactcaactatg | qRT-PCR |
| DPE2-R |  | gattaccaccagattcaa | qRT-PCR |
| EBP1 | At3g51800 | ccaatcgctcctccttaa | qRT-PCR |
| EBP1 |  | gaactcatctctgataactctc | qRT-PCR |
| GWD1/SEX1-F | At1g10760 | aagttggcaggttattagt | qRT-PCR |
| GWD1/SEX1-R |  | ttgtaggtctatcgtaggt | qRT-PCR |
| GWD2-F | At4g24450 | agtgtgataatggatgaag | qRT-PCR |
| GWD2-R |  | caatcgctgagaatagac | qRT-PCR |
| GWD3/PWD-F | At5g26570 | gacacatcaggagaatcaa | qRT-PCR |
| GWD3/PWD-R |  | cagtatcaggagcatcatt | qRT-PCR |
| HXK1-F | [AT4G29130](http://www.arabidopsis.org/servlets/TairObject?type=locus&id=127303) | ctacagaatgcgaagact | qRT-PCR |
| HXK1-R |  | taccagaggacagagaag | qRT-PCR |
| ISA3-F | At4g09020 | tggcatagacaacaaggt | qRT-PCR |
| ISA3-R |  | tggatggttacagttcagt | qRT-PCR |
| KIN10-F | At3g01090 | cagatggtatgctcagtaac | qRT-PCR |
| KIN10-R |  | tgcttcgttctctattatgc | qRT-PCR |
| KIN11-F | At3g29160 | gtttactttgccgggttac | qRT-PCR |
| KIN11-R |  | ccatcaaaaagaagaaagga | qRT-PCR |
| LDA-F | At5g04360 | aatgatgagactgttgag | qRT-PCR |
| LDA-R |  | aatgttggaagaagatgta | qRT-PCR |
| LHCA1-F | At3g54890 | tctcttcttccaagtctaa | qRT-PCR |
| LHCA1-R |  | attctgatacgaccaaca | qRT-PCR |
| LHCA2-F | At3g61470 | aataatggcatcatctctt | qRT-PCR |
| LHCA2-R |  | cagtcaacttcttcttca | qRT-PCR |
| LHCB1.1-F | At1g29920 | tgagccaagttctatctgt | qRT-PCR |
| LHCB1.1-R |  | ctctaccatccaccacaa | qRT-PCR |
| LHCB1.4-F | At2g34430 | gagtgaagttcggagaag | qRT-PCR |
| LHCB1.4-R |  | ccaagtagtccaatcctc | qRT-PCR |
| LSF1-F | At3g01510 | ggcattcagcaacttcac | qRT-PCR |
| LSF1-R |  | agcaacatcaccaactaca | qRT-PCR |
| LSF2-F | At3g10940 | gagaacgatgagtgtgatt | qRT-PCR |
| LSF2-R |  | agaagtgaagcaagaagatt | qRT-PCR |
| NAC1-F | AT1G56010 | aggagttatatgtagagac | qRT-PCR |
| NAC1-R |  | tgatgatgtagtgatgat | qRT-PCR |
| NAP-F | AT1G69490 | gaagaagagataatgatgatg | qRT-PCR |
| NAP-R |  | caggttgatgaagatgat | qRT-PCR |
| NPR1-F | AT1G64280 | attgccaaggattacgaagt | qRT-PCR |
| NPR1-R |  | tctcactctgctgctgta | qRT-PCR |
| ORE1/NAC2-F | At5g39610 | tacgaggcatcaagaatc | qRT-PCR |
| ORE1/NAC2-R |  | aggtagtgagttatgagttc | qRT-PCR |
| PHS1-F | At3g29320 | ggattggtggagaagacat | qRT-PCR |
| PHS1-R |  | gagccgcagattgatagt | qRT-PCR |
| PHS2-F | At3g46970 | agagattgacaagaggtt | qRT-PCR |
| PHS2-R |  | acacataagttagccattc | qRT-PCR |
| RPS6A | At4g31700 | agaagccaagaatgagag | qRT-PCR |
| RPS6A |  | ggtaagtgttgacataggt | qRT-PCR |
| SAG12-F | AT5G45890 | caagcactgatgaaggcagt | qRT-PCR |
| SAG12-R |  | tgcactctccagtgaacaca | qRT-PCR |
| SAG13 | AT2G29350 | gacaacataaggacgaactctg | qRT-PCR |
| SAG13 |  | tacgcaccgcttctttct | qRT-PCR |
| SAG2-F | AT5G60360 | tcttcttcttcttcttcttct | qRT-PCR |
| SAG2-R |  | aactactgatgataggattgt | qRT-PCR |
| SAUR36 | AT2g45210 | atgtctcgtctcactctc | qRT-PCR |
| SAUR36 |  | atcttcctcatcttcttatagc | qRT-PCR |
| SEN1-F | At4g35770 | gcttggacagagaatgag | qRT-PCR |
| SEN1-R |  | tgattgatacttgcgttga | qRT-PCR |
| SEX4-F | At3g52180 | tcttcttcttcttcttct | qRT-PCR |
| SEX4-R |  | gtcttggctatattcatc | qRT-PCR |
| TPS1-F | AT1G78580 | catcaggagaccgaagac | qRT-PCR |
| TPS1-R |  | tgttgttgttgttagagttagag | qRT-PCR |
| WRKY53-F | At4g23810 | catcatcgccaagattacat | qRT-PCR |
| WRKY53-R |  | ggtccttctaagcctctc | qRT-PCR |
| WRKY6-F | At1g62300 | ttctccttcgttaatctc | qRT-PCR |
| WRKY6-R |  | atcatcttcttcgtcaat | qRT-PCR |
| WRKY70-F | At3g56400 | aatgaaggaagaagacaa | qRT-PCR |
| WRKY70-R |  | gcctgatgatgataatct | qRT-PCR |
| RT98a-F | At1g10390 | atccgttgtcaaactacagagg | RT-PCR |
| RT98a-R |  | ggagacgagccaaaactaaagg | RT-PCR |
| RT98b-F | At1g59660 | ccaactcctgttacaaacccat | RT-PCR |
| RT98b-R |  | gtccaatctctcgccttccatc | RT-PCR |
| DRM1-qRT-F | At1g28330 | AGGAAGGAACATGTGGCACT | qRT-PCR |
| DRM1-qRT-R |  | GAGTCACCGCTGTACAACCA | qRT-PCR |
| DIN6-qRT-F | AT3G47340 | TCTCGACCCTGAATCCAAGATGAT | qRT-PCR |
| DIN6-qRT-R |  | GTCGTCAAAGGCTCTCCTTAGA | qRT-PCR |
| ATG8a-qRT-F | AT4G21980 | CAATTTGTATACGTGGTTCGT | qRT-PCR |
| ATG8a-qRT-R |  | AGCAACGGTAAGAGATCCAA | qRT-PCR |
| ATG8E-qRT-F | AT2G45170 | TCTTTAAGATGGACAACGATTTC | qRT-PCR |
| ATG8E-qRT-R |  | CTCAGCCTTTTCCACAATCA | qRT-PCR |
| SAG12-qRT-F1 | At5g45890 | TATTACAGGTTATGAGGATGTCCC | qRT-PCR |
| SAG12-qRT-R1 |  | ACCACATAGTCCTTGTTTATCC | qRT-PCR |
| WRKY53-qRT-F1 | AT4G23810 | CTTCTGCTAAACTGGTCATCC | qRT-PCR |
| WRKY53-qRT-R1 |  | CTCTCTCTGGGCTTATTCTCA | qRT-PCR |
| ACT2-F | At5g09810 | CTCATGAAGATTCTCACTGAG | RT-PCR |
| ACT2-R |  | ACAACAGATAGTTCAATTCCCA | RT-PCR |
| TIP41-F | At4g34270 | GTGAAAACTGTTGGAGAGAAGCAA | qRT-PCR |
| TIP41-R |  | TCAACTGGATACCCTTTCGCA | qRT-PCR |
