# Supplementary material for: Two Nucleoporin98 homologous genes jointly participate in the regulation of starch degradation to repress senescence in Arabidopsis
Source: BMC Plant Biol. 2020 Jun 26;20:292. doi: 10.1186/s12870-020-02494-1 (PMC7318766; doi:10.1186/s12870-020-02494-1)
Supplement: Supplementary file 2 — Additional file 2:Figure S1. Protein domains in Nup98a and Nup98b of Arabidopsis thaliana. [file 12870_2020_2494_MOESM2_ESM.docx]

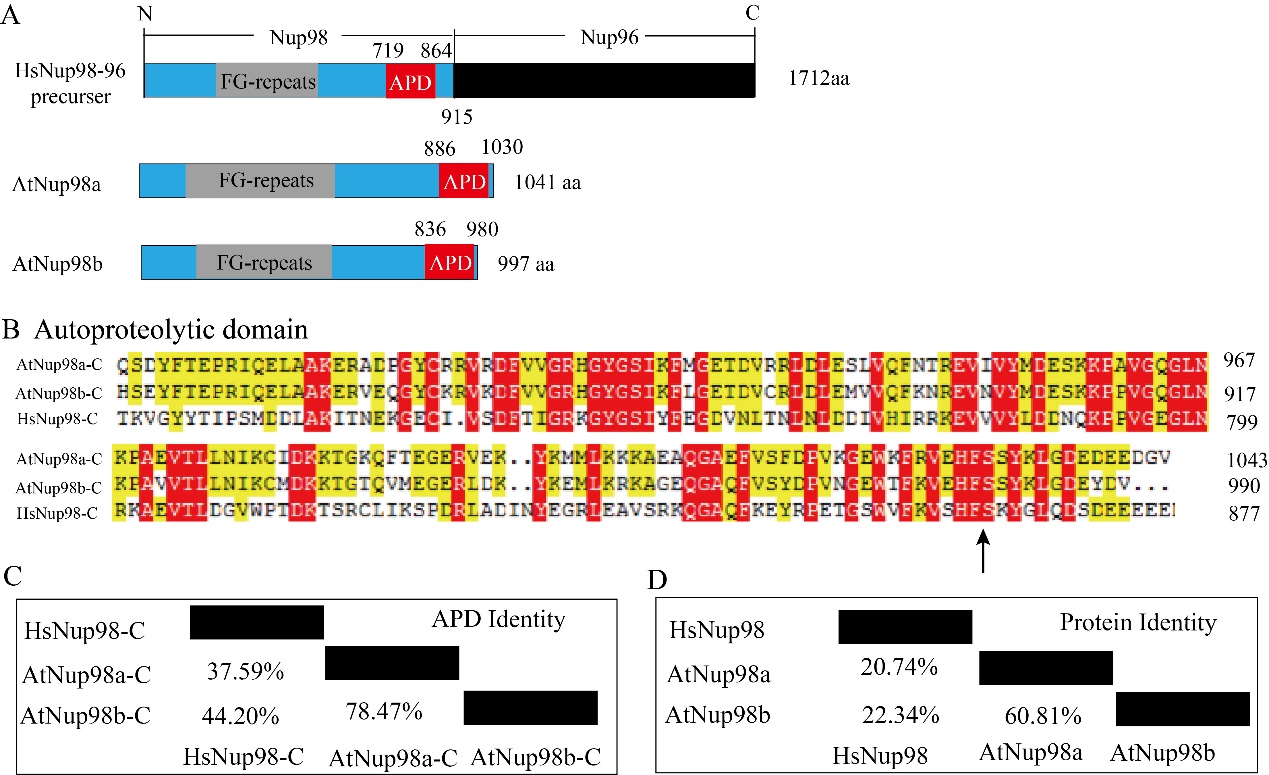


**Figure S1. Protein domains in Nup98a and Nup98b of *Arabidopsis thaliana*.** A, Sketches of Nup98-96 proteins from *Homo sapiens* HsNup98-96 (NM_016320.4) and two homologs from *Arabidopsis* *thaliana* Nup98a (At1g10390) and Nup98b (At1g59660). APD, autoproteolytic domains. FG, phenylalanine and glycine repeats. B, Alignment of the autoproteolytic domains of HsNup98-96, At Nup98a and At Nup98b. The arrow indicates the conserved HFS cleavage site. C, Overall identity of the autoproteolytic domains of HsNup98-96, At Nup98a and At Nup98b. D, Overall identity of the proteins of HsNup98-96, At Nup98a and At Nup98b.
