# Supplementary figures and images for: Two Nucleoporin98 homologous genes jointly participate in the regulation of starch degradation to repress senescence in Arabidopsis
Source: BMC Plant Biol. 2020 Jun 26;20:292. doi: 10.1186/s12870-020-02494-1 (PMC7318766; doi:10.1186/s12870-020-02494-1)

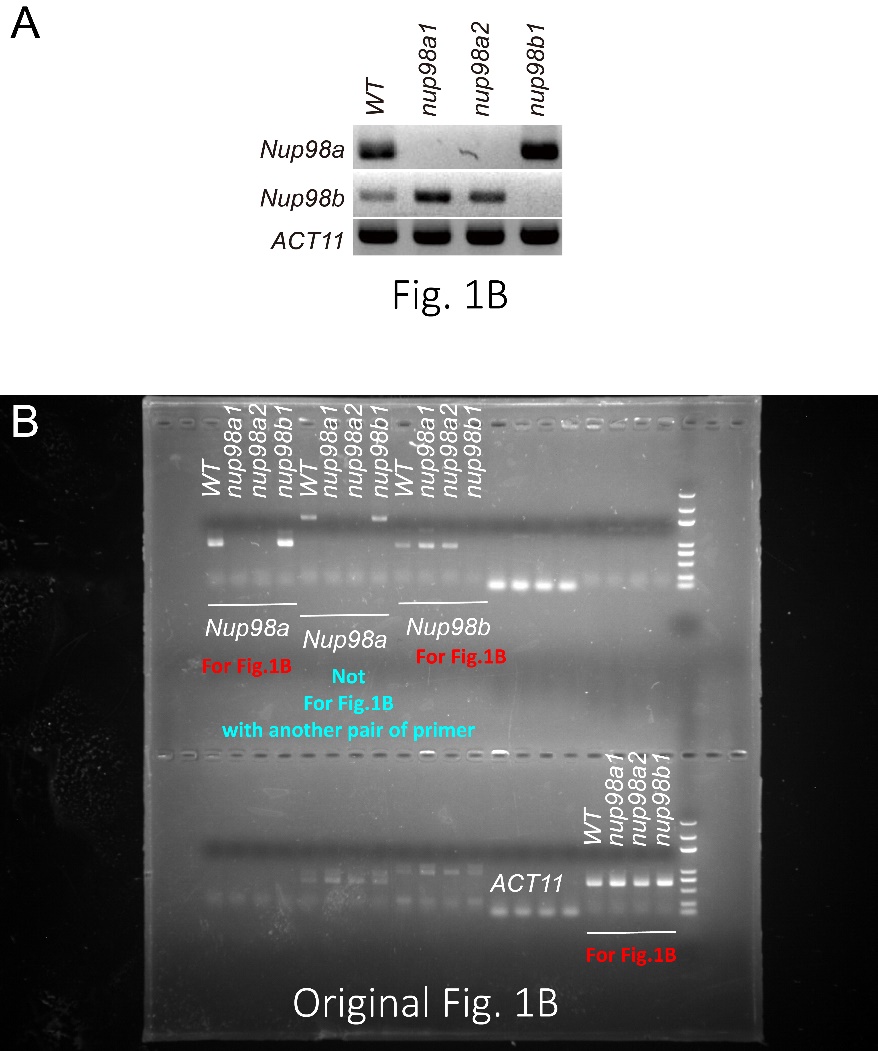


**Figure S2. The original photograph of the gel in Figure 1B.** A, Fig.1B; B, The original photo.

Supplement: Supplementary file 3 — Additional file 3:Figure S2. The original photograph of the gel in Fig. 1b. [file 12870_2020_2494_MOESM3_ESM.docx]
