# Supplementary material for: Two Nucleoporin98 homologous genes jointly participate in the regulation of starch degradation to repress senescence in Arabidopsis
Source: BMC Plant Biol. 2020 Jun 26;20:292. doi: 10.1186/s12870-020-02494-1 (PMC7318766; doi:10.1186/s12870-020-02494-1)
Supplement: Supplementary file 4 — Additional file 4:Figure S3. The nup98a1, nup98b1 double mutant showed pleiotropic phenotypes in various organs. [file 12870_2020_2494_MOESM4_ESM.docx]

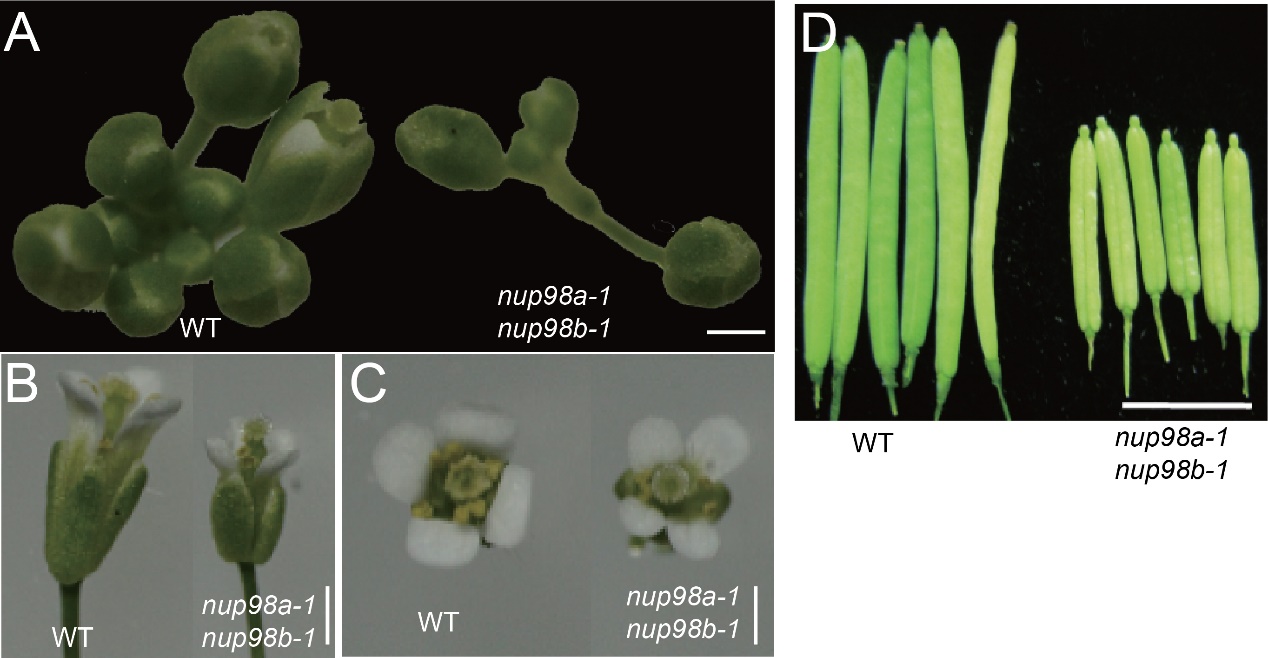


**Figure S3. The *nup98a1*, *nup98b1* double mutant showed pleiotropic phenotypes in various organs.** The seeds of mutant and WT were sown in soil after low temperature treatment for 3 days on wet filter paper and grew in long day conditions. **A**, A top view of Inflorescence; B, A side view of flowers; C, A top view of flowers; D, Siliques. Scale bars = 2 mm. All the images are our own data.
