# Supplementary material for: Two Nucleoporin98 homologous genes jointly participate in the regulation of starch degradation to repress senescence in Arabidopsis
Source: BMC Plant Biol. 2020 Jun 26;20:292. doi: 10.1186/s12870-020-02494-1 (PMC7318766; doi:10.1186/s12870-020-02494-1)
Supplement: Supplementary file 5 — Additional file 5:Figure S4. The nup98a1, nup98b1 double mutant showed a senescent and sterile phenotype. [file 12870_2020_2494_MOESM5_ESM.docx]

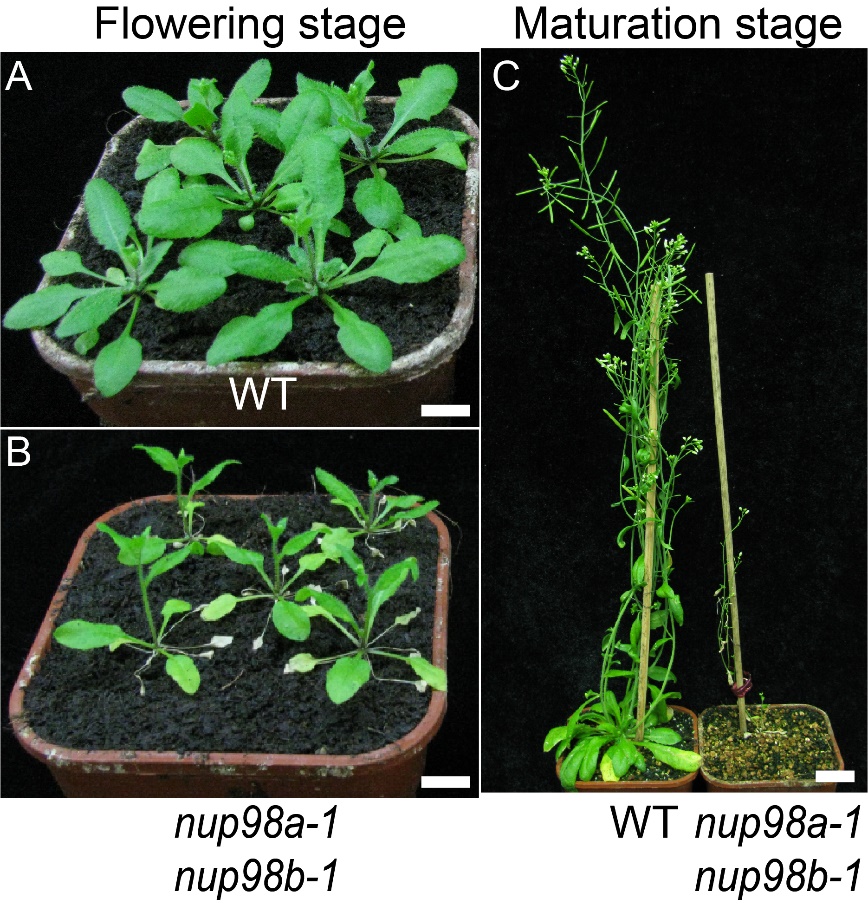


**Figure S4. The *nup98a1*, *nup98b1* double mutant showed a senescent and sterile phenotype.** The seeds of mutant and WT were sown in soil after low temperature treatment for 3 days on wet filter paper and grew in long day conditions. **A** and **B**, Plants at early stage of flowering. Plants grew in dishes supplemented with MS and sucrose for one week, and then transplanted in soil. **C**, Plants at mature stage. All the images are our own data.
