# Supplementary material for: Two Nucleoporin98 homologous genes jointly participate in the regulation of starch degradation to repress senescence in Arabidopsis
Source: BMC Plant Biol. 2020 Jun 26;20:292. doi: 10.1186/s12870-020-02494-1 (PMC7318766; doi:10.1186/s12870-020-02494-1)
Supplement: Supplementary file 6 — Additional file 6:Figure S5. Senescent phenotypes were specific to the nup98a1, nup98b1 double mutant compared with mutants of other nucleoporins. [file 12870_2020_2494_MOESM6_ESM.docx]

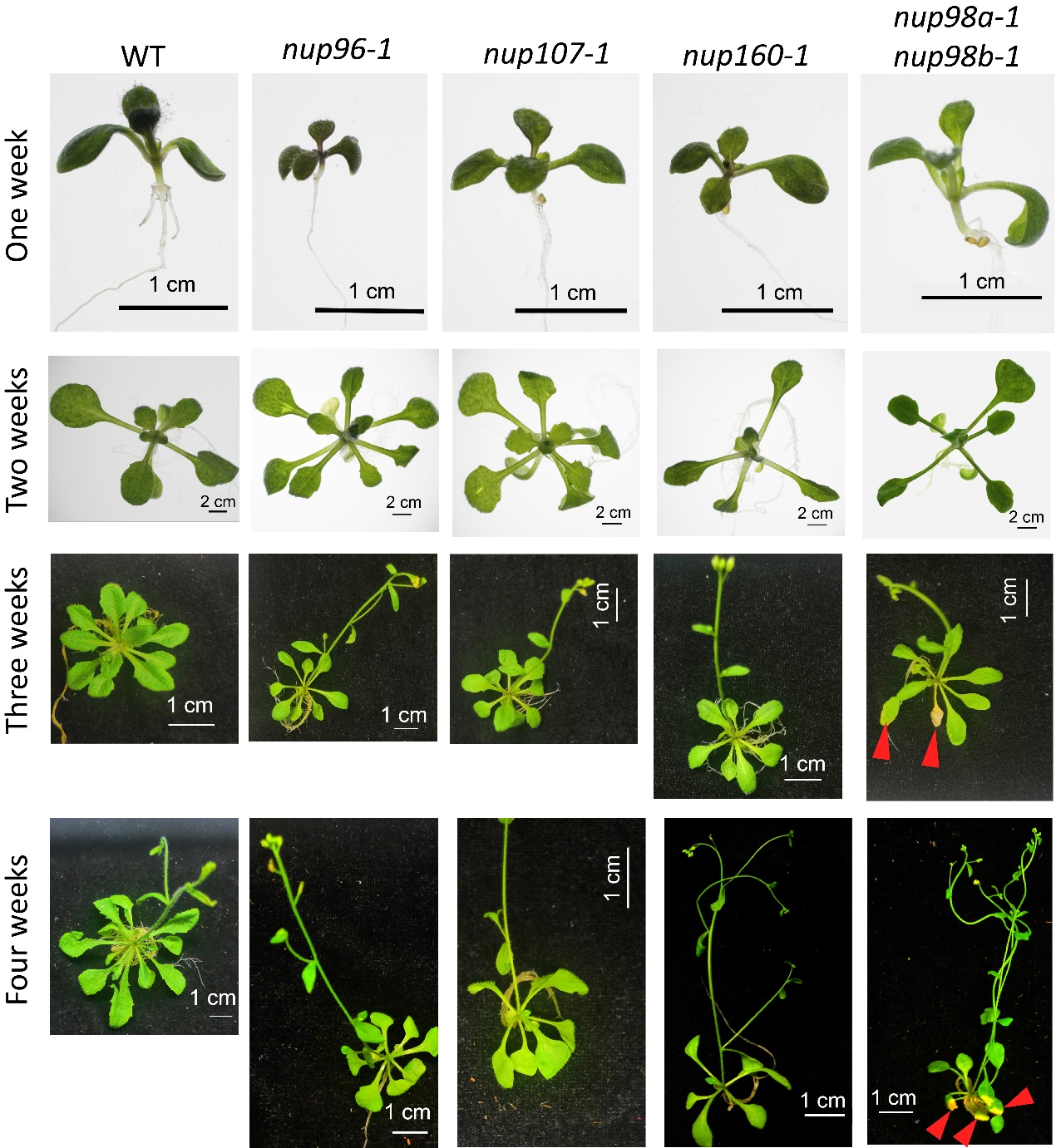


**Figure S5. Senescent phenotypes were specific to the *nup98a1*, *nup98b1* double mutant compared with mutants of other nucleoporins.** The data showed that the mutants of nucleoporin genes, *Nup96*, *Nup107*, and *Nup160*, did not exhibit senescence characteristics even though plants were in bloom, while the *nup98a-1 nup98b-1* mutant appeared senescence symptom (yellow leaves, red arrows) from three weeks after germination. All plants grew in long day conditions. All the images are our own data.
