# Supplementary material for: Two Nucleoporin98 homologous genes jointly participate in the regulation of starch degradation to repress senescence in Arabidopsis
Source: BMC Plant Biol. 2020 Jun 26;20:292. doi: 10.1186/s12870-020-02494-1 (PMC7318766; doi:10.1186/s12870-020-02494-1)
Supplement: Supplementary file 7 — Additional file 7:Figure S6. Molecular network of senescence initiation in plants. [file 12870_2020_2494_MOESM7_ESM.docx]

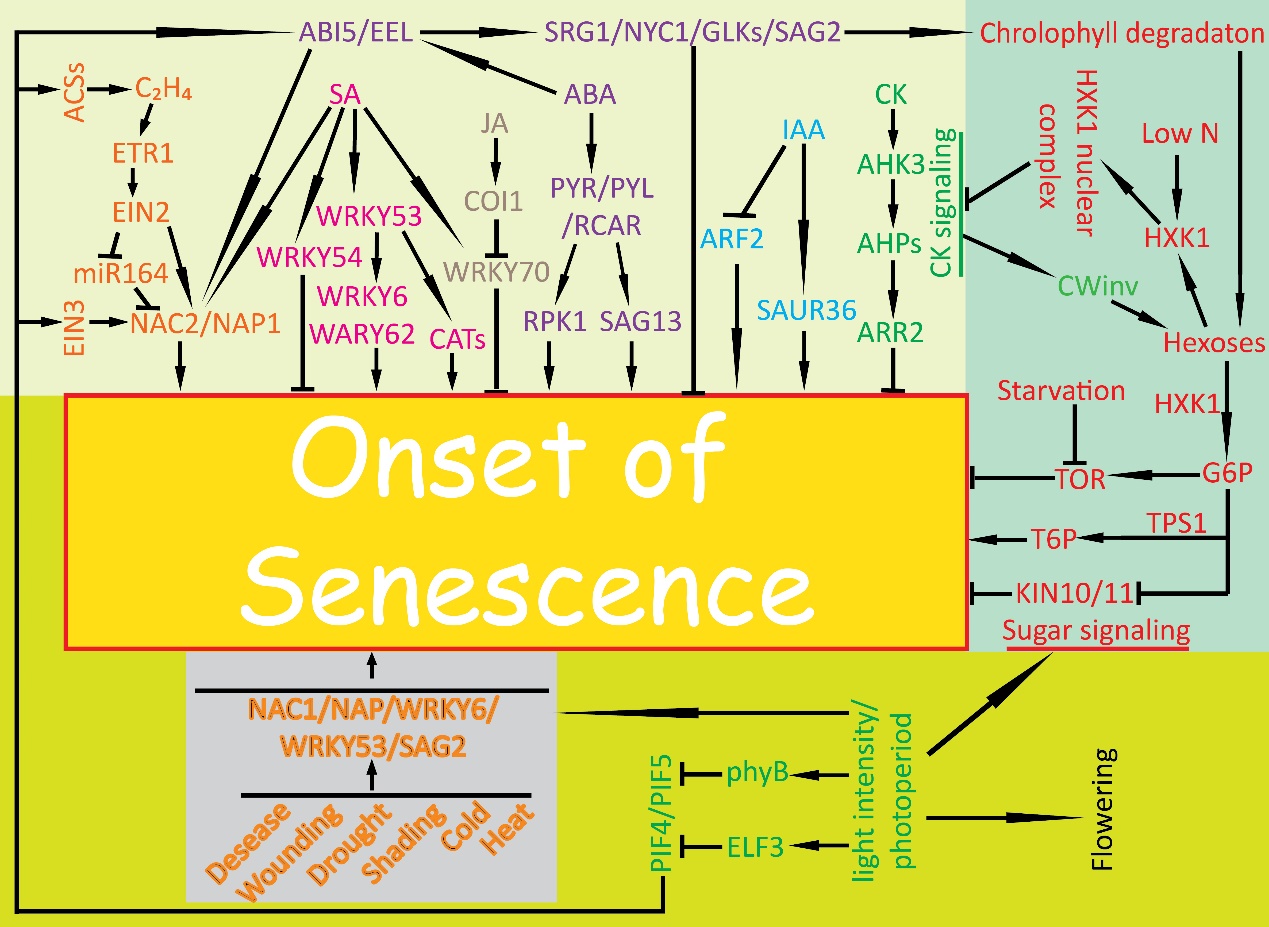


**Figure S6. Molecular network of senescence initiation in plants.** The summary is based on previous publications [^1-12^](#_ENREF_1), but not limited these publications. Generally, initiation of senescence includes the hormone (light yellow background), sugar metabolism (light green background), and light and photoperiod pathway (green-yellow background), and stress pathways (grey background). These pathways can induce senescence at different developmental stages (yellow background). Genes and metabolites in the same pathway are indicated by the same color. Due to space limitations, this summary model does not include all genes identified to date. Abbreviations: ABA, abscisic acid; ABI, ABA INSENSITIVE; AHK, *ARABIDOPSIS* HISTIDINE KINASE; ACS, AMINOCYCLOPROPANE-1-CARBOXYLATE (ACC) SYNTHASE; AHP, *ARABIDOPSIS THALIANA* HISTIDINE PHOSPHOTRANSFER PROTEINS; ARF, AUXIN RESPONSE FACTOR; ARR, ARABIDOPSIS RESPONSE REGULATOR; C_2_H_4_, ethylene ; CAT, CATALASE; CK, cytokinin; COI1, CORONATINE INSENSITIVE 1; CWINV, CELL WALL INVERTASE; EEL, ENHANCED EM LEVEL; EIN, ETHYLENE INSENSITIVE; ELF, EARLY FLOWERING; ETR, ETHYLENE RESPONSIVE; G6P，6-phosphoric acid glucose; GLK, Golden 2-like Transcription factor; HXK1, HEXOKINASE 1; KIN, PROTEIN KINASE10; N, nitrogen; NAC，NAM, ATAF, AND CUC; NAP, NAC-LIKE PROTEIN; NYC, NONYELLOW COLORING; ORE, ORESARA; PHYB, phytochrome B; PIF, PHYTOCHROME INTERACTING FACTOR; PYR/PYL/RCAR, PYRABACTIN RESISTANCE/PYR1-LIKE ORREGULATORY COMPONENT OF ABA RECEPTOR; SAG, SENESCENCE-ASSOCIATED GENE; SAUR, SMALL AUXIN UPREGULATED; SGR, STAYGREEN; T6P, TREHALOSE-6-PHOSPHATE; TOR, TARGET OF RAPAMYCIN; TPS1, TREHALOSE-6-PHOSPHATE SYNTHASE 1; WRKY, WRKY DNA-BINDING PROTEIN. Arrowheads indicate promoting effect while short bars indicate inhibiting effect on senescence. All the images are our own data.
