# Supplementary material for: Two Nucleoporin98 homologous genes jointly participate in the regulation of starch degradation to repress senescence in Arabidopsis
Source: BMC Plant Biol. 2020 Jun 26;20:292. doi: 10.1186/s12870-020-02494-1 (PMC7318766; doi:10.1186/s12870-020-02494-1)
Supplement: Supplementary file 8 — Additional file 8:Figure S7. A simplefied pathway of starch degradation in chloroplasts. [file 12870_2020_2494_MOESM8_ESM.docx]

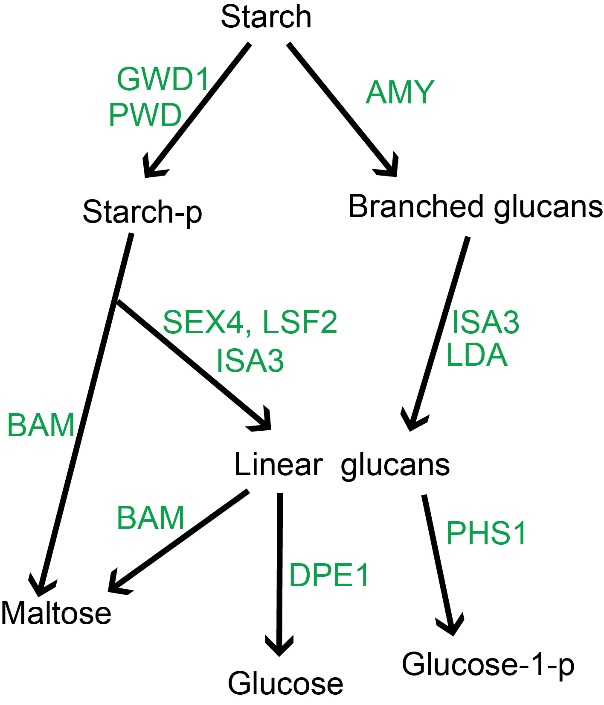


**Figure S7. A simplefied pathway of starch degradation in chloroplasts.** Starch is degraded into maltose, glucose, or glucose -1-phospholate in chloroplast, and these three monosaccharides are exported from chloroplast into cytoplasm to provide energy source for cells. Black words indicate saccharides, while green words represent enzymes degrading starch (only main enzymes showed). GWD, α-glucan, water dikinase; PWD, phosphoglucan, water dikinase; AMY, α-amylase; BAM, β-amylase; SEX4, STARCH EXCESS 4 (phosphoglucan phosphatase); LSF2, LIKE SEX FOUR2 (phosphoglucan phosphatase); DPE, disproportionating enzyme; ISA3, iso-amylase3; LDA, limit-dextrinase; PHS, α-glucan phosphorylase. These degradation steps happen in chloroplasts, and the products of maltose, glucose and glucose-1-p would be exported from chloroplasts into cytoplasm.
