# Supplementary material for: Two Nucleoporin98 homologous genes jointly participate in the regulation of starch degradation to repress senescence in Arabidopsis
Source: BMC Plant Biol. 2020 Jun 26;20:292. doi: 10.1186/s12870-020-02494-1 (PMC7318766; doi:10.1186/s12870-020-02494-1)
Supplement: Supplementary file 9 — Additional file 9:Figure S8. Expression analysis of genes related to photosynthesis and sugar metabolism in the nup98a1, nup98b1 double mutant. [file 12870_2020_2494_MOESM9_ESM.docx]

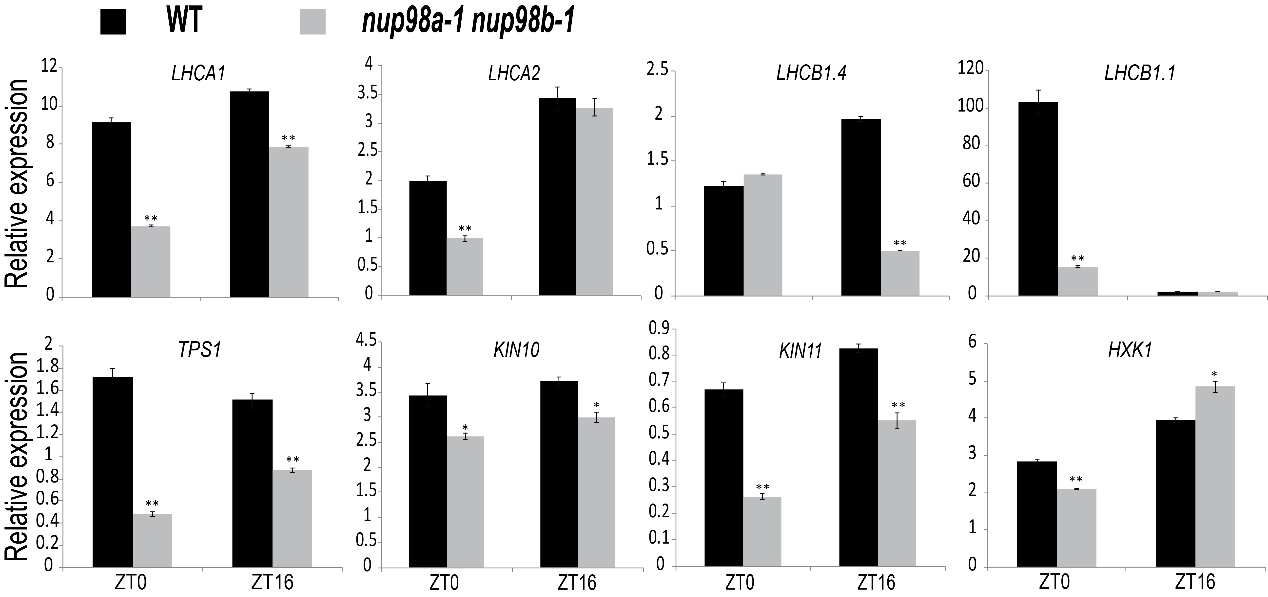


**Figure S8. Expression analysis of genes related to photosynthesis and sugar metabolism in the *nup98a1*, *nup98b1* double mutant.** The *nup98a-1 nup98b-1* double mutant and WT plants were grown in petri dishes in long day conditions for 14 days, and then harvested at ZT0 and ZT16 for gene expression analysis. All qPCR measurements were repeated at least three times, in triplicate. Gene expression measurements were normalized to the control At4g34270 and expressed as a relative expression value. Student’s t test was used to statistically analyze the data. An * indicates measurements that were significantly (**P* < 0.05; ***P* <0.01) different from the control. Error bars indicate ± SD of the mean.
