# Supplementary material for: Two Nucleoporin98 homologous genes jointly participate in the regulation of starch degradation to repress senescence in Arabidopsis
Source: BMC Plant Biol. 2020 Jun 26;20:292. doi: 10.1186/s12870-020-02494-1 (PMC7318766; doi:10.1186/s12870-020-02494-1)
Supplement: Supplementary file 10 — Additional file 10:Figure S9. Exogenous sucrose reduces early senescence in the nup98a1, nup98b1 double mutant plants. [file 12870_2020_2494_MOESM10_ESM.docx]

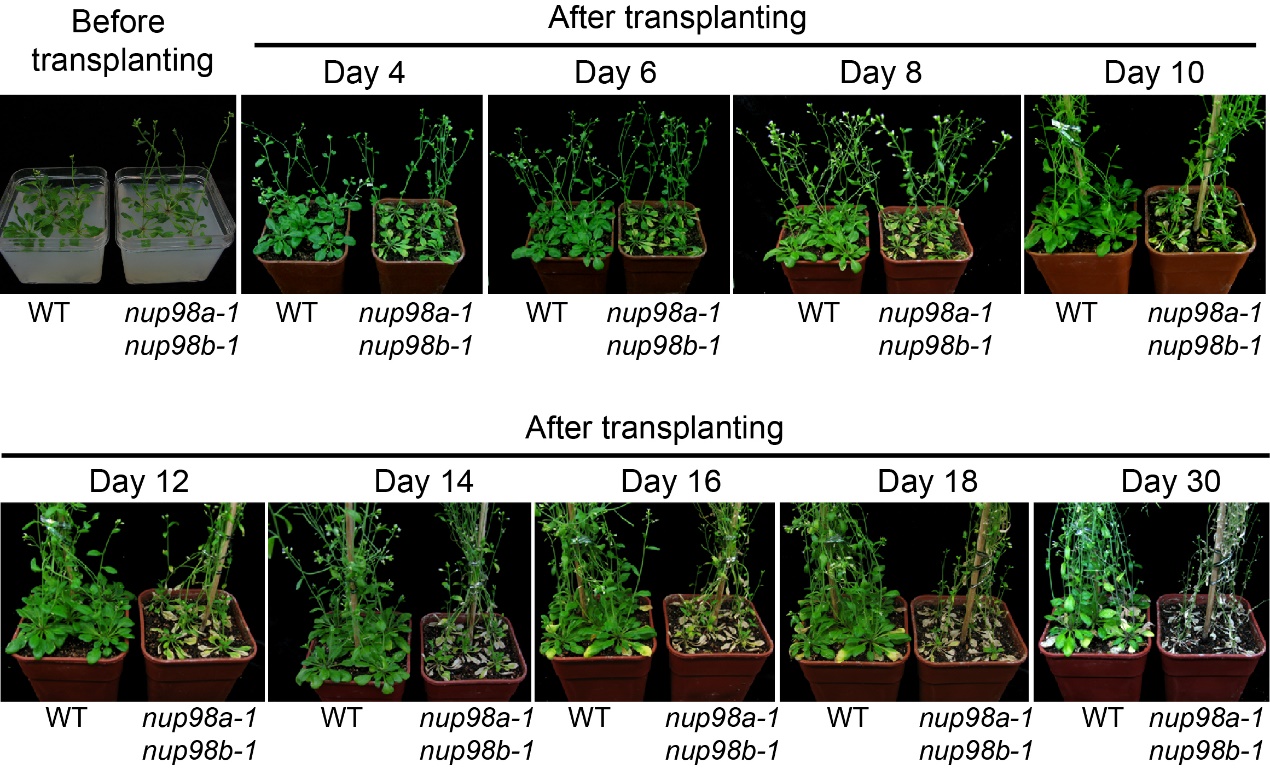


**Figure S9. Exogenous sucrose reduces early senescence in the *nup98a1*, *nup98b1* double mutant plants.** Medium is MS (Murashige & Skoog) basal nutrients and 3% sucrose. The seeds of WT and the *nup98a-1 nup98b-1* double mutant were sown on MS medium with sucrose. After stratification, plants were grown under long day conditions. After inflorescences emerged, plants were transplanted to soil. Senescence symptoms appeared 4 days later in the double mutant leaves, about 12 days earlier than the wild type control. All the images are our own data.
