# Supplementary material for: Two Nucleoporin98 homologous genes jointly participate in the regulation of starch degradation to repress senescence in Arabidopsis
Source: BMC Plant Biol. 2020 Jun 26;20:292. doi: 10.1186/s12870-020-02494-1 (PMC7318766; doi:10.1186/s12870-020-02494-1)
Supplement: Supplementary file 11 — Additional file 11:Figure S10. Phenotype analysis of the nup98a1, nup98b1 double mutant compared with WT grown on different mediums. [file 12870_2020_2494_MOESM11_ESM.docx]

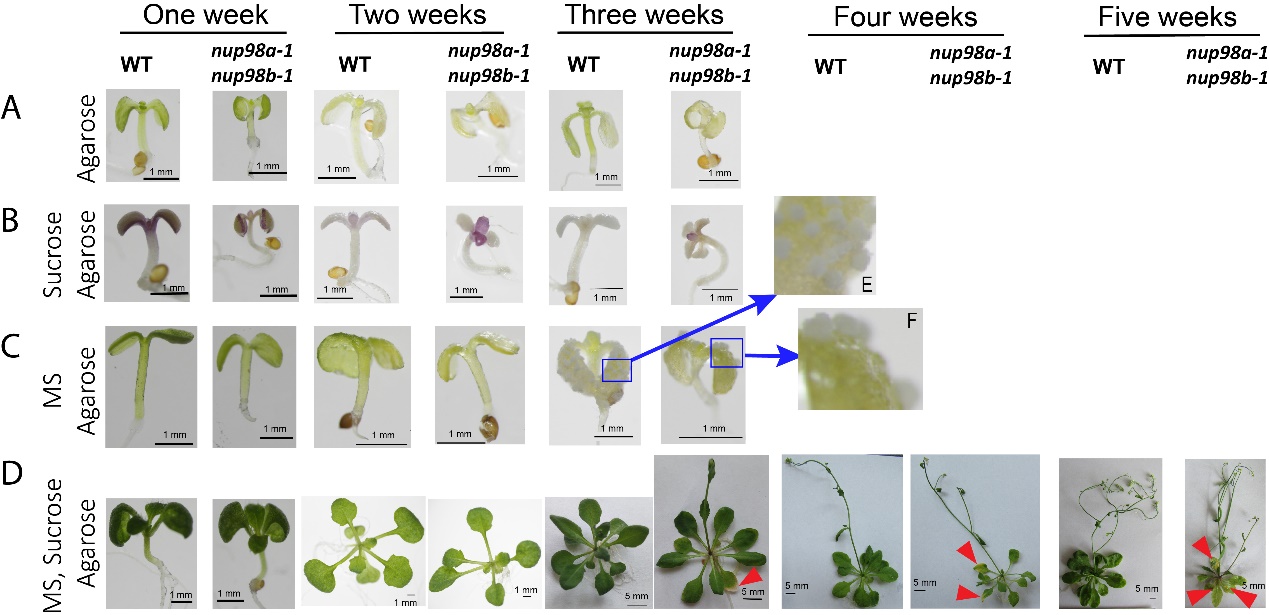


**Figure S10.** **Phenotype analysis of the *nup98a1*, *nup98b1* double mutant compared with WT grown on different mediums.** Medium is MS (Murashige & Skoog) basal nutrients and 3% sucrose. Row A, agarose medium. Row B, agarose plus sucrose. Row C, agarose plus MS basal nutrients. Row D, agarose plus sucrose and MS basal nutrients. Plants cannot fulfil the life cycle and die in about three weeks after germination in medium A, B, and C, whereas plants in medium D can go through the life cycle. There were white callus-like structures found on the leaf surfaces of three week-seedlings on medium C (blue arrow, E and F). The *nup98a-1* *nup98b-1* double mutant displayed senescence leaves (red arrow heads) early than WT (from week three) as Figure 5 showed. We also observed that the seedlings of 25% of WT or 8% of the *nup98a-1* *nup98b-1* double mutant on medium C can grow normal more or less until three weeks after germination. All experiments were repeated at least twice, and one representative photo each was showed here. All the images are our own data.
